# Germline *TTN* variants are enriched in *PTEN*-wildtype Bannayan–Riley–Ruvalcaba syndrome

**DOI:** 10.1038/s41525-017-0039-y

**Published:** 2017-12-18

**Authors:** Lamis Yehia, Ying Ni, Charis Eng

**Affiliations:** 1 Genomic Medicine Institute, Cleveland Clinic, Cleveland, OH 44195 USA; 2 Lerner Research Institute, Cleveland Clinic, Cleveland, OH 44195 USA; 3 Department of Pathology, Case Western Reserve University, Cleveland, OH 44106 USA; 4 Center for Clinical Genomics, Cleveland Clinic, Cleveland, OH 44106 USA; 5 Taussig Cancer Institute, Cleveland Clinic, Cleveland, OH 44195 USA; 6 Department of Genetics and Genome Sciences, Case Western Reserve University School of Medicine, Cleveland, OH 44106 USA; 7 Germline High Risk Focus Group, CASE Comprehensive Cancer Center, Case Western Reserve University, Cleveland, OH 44106 USA

## Abstract

Bannayan–Riley–Ruvalcaba syndrome (BRRS) is a rare congenital disorder classically characterized by macrocephaly in combination with intestinal hamartomatous polyposis, vascular malformations, lipomas, and genital lentiginosis. Germline *PTEN* mutations have been reported in up to 60% of BRRS patients. The remaining cases are of unknown genetic etiology. We exome-sequenced 35 unrelated *PTEN-*wildtype patients with classic presentation of BRRS and identified *TTN* germline missense variants in 12/35 (34%) patients. *TTN* encodes TITIN, a key structural and functional muscle protein. Exome and *TTN-*targeted sequencing in an additional unrelated series of 231 BRRS-like patients revealed 37 (16%) additional patients with germline *TTN* variants. All variants were predicted to be deleterious and equally distributed between the A-band and I-band protein domains. Rare *TTN* variants (MAF ≤ 0.0001) are enriched in classic BRRS patients compared to BRRS-like (OR = 2.7, 95% CI 1.21-5.94, *p* = 1.6 × 10^-2^) and multiple population controls (OR = 2.2, 95% CI 1.01-4.20, *p* = 4.7 × 10^-2^). Germline *TTN* mutations of different genotypes, inheritance patterns, and protein domain enrichment have been identified in multiple cardiac and/or skeletal muscular disorders. Functional interrogation of I-band variant p.Cys5096Arg identified in one of our classic BRRS patients, using CRISPR-Cas9 genome-edited cell lines, reveals an increased growth and lack of contact inhibition phenotype associated with increased levels of or phosphorylation of focal adhesion kinase (FAK) in mutant cells. These findings suggest that TITIN could play a role in overgrowth-relevant pathways and phenotypes. In summary, our observations suggest *TTN* as a candidate predisposing gene in classic *PTEN-*wildtype BRRS patients, perhaps suggesting this syndrome join the growing list of Titinopathies.

## Rare disease: New gene linked to Bannayan-Riley-Ruvalcaba syndrome

*TTN* variants seem to explain many cases of a rare hereditary condition previously linked only to mutations in the unrelated gene *PTEN*. Charis Eng and team from the Cleveland Clinic, Ohio, USA, sequenced the protein-coding DNA from 35 patients with Bannayan-Riley-Ruvalcaba syndrome (BRRS), a disease characterized by large head size, tumors and other overgrowth problems. All of these individuals showed no mutations in *PTEN*, a tumor suppressor that explains 60% of BRRS cases. However, one-third had variants in *TTN*, which encodes a protein called TITIN that’s involved in muscle elasticity. Additional testing revealed more BRRS-like patients with *TTN* variants, and lab experiments indicated one possibility of how *TTN* variants lead to increased cellular growth. The authors suggest BRRS could be considered a “titinopathy” along with other cardiac and skeletal diseases caused by *TTN* mutations.

## Introduction

Bannayan–Riley–Ruvalcaba syndrome (BRRS [MIM 153480]) is a congenital disorder classically characterized by macrocephaly in combination with intestinal hamartomatous polyposis, vascular malformations, lipomas, hemangiomas, and genital freckling.^1^ The true prevalence of BRRS is unknown, although it is generally considered rare. Clinical features manifest neonatally and are often variable amongst individuals.^2^ In addition to the cardinal clinical features, other reported phenotypes include high birth weight, developmental delay, mild-to-severe mental retardation, delayed psychomotor development, muscle hypotonia, lipid storage myopathy, joint hyperextensibility, pectus excavatum, and scoliosis.^1,3–5^ Clinically, the differential diagnosis includes other genetic disorders with overlapping phenotypes of macrocephaly, gastrointestinal polyposis, and benign tumors, such as Cowden syndrome (CS; MIM 158350), juvenile polyposis syndrome (JPS; MIM 174900), Peutz–Jeghers syndrome (PJS; MIM 175200), and neurofibromatosis type 1 (NF1; MIM 162200). However, among these disorders, BRRS is considered to be allelic only to CS, as both syndromes have been associated with germline mutations in the tumor suppressor gene phosphatase and tensin homolog (*PTEN* [MIM 158350]) at 10q23.3, the latter excluded as a candidate locus for the other overlapping syndromes.^6–9^

Germline *PTEN* mutations have been reported in up to 60% of BRRS patients.^10–14^ Among those who remain mutation negative, approximately 10% were found to harbor large deletions of *PTEN*.^14^ Such BRRS patients with germline *PTEN* mutations belong to the PTEN hamartoma tumor syndrome (PHTS), which also includes *PTEN*-related Cowden, Cowden-like, Proteus, and Proteus-like syndromes.^15^ Although these conditions are inherited autosomal dominantly, a recent study showed that de novo* PTEN* mutations occur in at least 10% of molecularly tested PHTS probands,^11,12,16–18^ including *PTEN-*related BRRS.^19^

However, the underlying genetic causes remain undetermined in the subset of BRRS patients without alterations in *PTEN*, and hence additional predisposing genes must exist in these patients. Currently, all BRRS patients are clinically managed as though they have *PTEN* mutations, even if they are constitutionally wildtype for *PTEN*. In this context, the identification of additional BRRS-relevant genes could improve molecular diagnosis and especially predictive testing, risk assessment, genetic counseling and clinical management of patients. Therefore, we sought to identify other predisposition gene(s) for BRRS via an exome sequencing approach, and subsequently, to characterize the functional consequences of the prioritized gene(s).

## Results

### Identification of candidate predisposition genes in *PTEN-*wildtype BRRS

We identified 14 eligible unrelated BRRS patients based on wildtype *PTEN* mutation status and phenotypic burden. All patients were male and presented with penile freckling in addition to other classic BRRS features (Supplementary Table 1). Ages at consent ranged from 1 to 68 years (median = 36 ± 19 years). We performed exome sequencing on germline genomic DNA from peripheral blood leukocytes of these patients. Initial filtering and variant prioritization identified an average of 37 ± 10 variants (range: 21–54) per patient that occur in conserved genomic regions and that have not been observed in public databases (dbSNP137/8, NHLBI-ESP6500, and 1000 Genomes) with a cut-off minor allele frequency (MAF) of 0.0005 (0.05%).

To prioritize the filtered variants, we first looked for shared genes that were mutated in at least two patients. Using this approach, we identified 11 genes, with the highest number of variants observed for *TTN* (*n* = 9, MIM 188840) and *TRAP1* (*n* = 3, MIM 606219). The remaining nine genes showed variants in two patients each and included *DNAH11* (MIM 603339), *FRAS1* (MIM 607830), *FRMD6* (MIM 614555), *HEXA* (MIM 606869), *ITGA7* (MIM 600536), *LTA4H* (MIM 151570), *PXDN* (MIM 605158), *SEZ6L2* (MIM 616667), and *THBS2* (MIM 188061) (Supplementary Table 2).

To validate our findings, we then performed exome sequencing on an additional series of unrelated *PTEN-*wildtype classic BRRS patients (*n* = 21). This patient series included both males (*n* = 17) and females (*n* = 4) with ages at consent ranging from 1 to 72 years (median = 16 ± 23 years) (Supplementary Table 3). Targeted analysis of the previously prioritized genes revealed four additional *TTN* variants and no further variants in the other 11 genes. Collective analysis of both BRRS series (*n* = 35) revealed six other genes with variants occurring in at least three patients: *AK9* (*n* = 4, MIM 615358), *ANKAR* (*n* = 3, MIM 609803), *CDH24* (*n* = 3, HGNC 14265), *ITPR3* (*n* = 3, MIM 147267), *SSPO* (*n* = 3, MIM 617356), and *STARD9* (*n* = 3, MIM 614642) (Supplementary Table 4).

### Germline *TTN* variants are enriched in patients with classic BRRS features

We prioritized *TTN* for further downstream analysis because overall, exome sequencing of (the above) 35 unrelated BRRS probands revealed the existence of *TTN* variants in 12 (34%) patients (Table 1). All variants were validated by PCR-based region-specific Sanger sequencing and only one patient (CCF07445) had two variants identified. All variants were also predicted to affect highly conserved amino acid residues and to be damaging according to in silico predictions.^20–22^ Moreover, all variants that we identified in BRRS and that have been previously reported in NHLBI-ESP6500 and/or ExAC populations had a MAF ≤ 0.0005 (Table 2). None of these variants were reported in the 1000G database. Additionally, in order to identify whether the *TTN* variants segregated with the BRRS phenotype, we searched our clinical database for other recruited affected and unaffected family members of the sequenced probands, and with available DNA. We identified one eligible family satisfying these criteria, consisting of a trio with the proband’s father also being affected (Supplementary Fig. 1). Indeed, targeted genotyping using Sanger sequencing identified the same germline *TTN* variant in the affected father, whereas the unaffected mother showed wildtype genotype.

**Table 1:** Clinical and demographic characteristics of 12 unrelated BRRS probands with germline *TTN* variants

Subject	Age at consent	Gender	Macrocephaly	Penile freckling	Neuro-psychological	Benign overgrowths and skin features	Other phenotypes and incidental findings
CCF00162	33	M	Yes (59 cm)	Yes	Unknown	Lipoma, hemangioma (NOS)	Prominent Schwalbe’s lines
CCF02011	1	M	Yes (59 cm)	Yes	Developmental delay/ASD	Unknown	Unknown
CCF06949	51	M	Yes (58.5 cm)	Yes	Developmental delay/ASD	Skin hemangioma	Obsessive compulsive disorder
CCF07265	68	M	Yes (61 cm)	Yes	Unknown	Mucocutaneous lip pigmentation	Prostate cancer (age 65)
CCF06673	15	M	No	Yes	Unknown	Skin fibroma	Follicular variant papillary thyroid cancer (age 13)
CCF07445	38	M	Yes (59.2 cm)	Yes	Unknown	Trichilemmoma, acral keratoses, skin tag	Unknown
CCF01021	31	M	Yes (63.5 cm)	Yes	Mental retardation	Skin hemangioma, papillomatous papules (mucosa)	Hypotonia, hydrocephalus
CCF02423	45	M	Yes (59 cm)	Yes	Developmental delay/ASD	Unknown	Melanosis coli, melanoma in situ (age 41), renal clear cell cancer (age 42)
CCF02153	3	M	Yes (57.2 cm)	Yes	Developmental delay/ASD	Not observed	Unknown
CCF06523	26	M	No (54 cm)	Yes	Unknown	Lipoma	Unknown
CCF02289	46	M	No (55.5 cm)	Yes	Unknown	Lipoma	Unknown
CCF08133	71	M	Yes (59.6 cm)	Yes	Unknown	Lipoma, visceral hemangioma, goiter, benign breast disease	Follicular variant papillary thyroid cancer (age 68), renal cell cancer (age 64)

*M* male, *cm* centimeters, *ASD* autism spectrum disorder,* NOS* not otherwise specified

**Table 2:** *TTN* germline variants identified in 12/35 (34%) unrelated classic BRRS patients

Subject	Genomic position^a^	Exon	Variant	Protein alteration	Region	In silico predictions^b^	Protein stability^c^	NHLBI-ESP^d^	1000G^d^	ExAC^d^
CCF00162	Chr. 2: 179446909	315	c.66187G>C	p.V22063L	A-band	Damaging	Decreased (ΔΔ*G* = −1.30)	0	0	12/119044 (0.0001008), 0 hom
CCF02011	Chr. 2: 179543195	144	c.33856G>A	p.E11286K	I-band	Damaging	Decreased (ΔΔ*G* = −0.81)	T = 1/C = 11927 (0.000084)	0	32/55660^e^ (0.0005749), 0 hom
CCF06949	Chr. 2: 179574497	99	c.28549G>A	p.V9517M	I-band	Damaging	Decreased (ΔΔ*G* = −2.09)	0	0	0
CCF07265	Chr. 2: 179413865	339	c.92488G>A	p.V30830I	A-band	Damaging	Decreased (ΔΔ*G* = −1.09)	T = 2/C = 12176 (0.000164)	0	3/120482 (2.49e-05), 0 hom
CCF06673	Chr. 2: 179584872	81	c.23497G>C	p.G7833R	I-band	Damaging	Decreased (ΔΔ*G* = −1.32)	0	0	5/120520 (4.149e-05), 0 hom
CCF01021	Chr. 2: 179599265	52	c.15286T>C	p.C5096R	I-band	Damaging	Decreased (ΔΔ*G* = −1.47)	0	0	0
CCF02423	Chr. 2: 179571284	102	c.29317G>A	p.A9773T	I-band	Damaging	Decreased (ΔΔ*G* = −0.73)	T = 2/C = 12092 (0.000165)	0	7/120746 (5.797e-05), 0 hom
CCF07445	Chr. 2: 179442383 Chr. 2: 179396767	323 358	c.68770G>A c.104575C>T	p.A22924T p.R34859W	A-band M-band	Damaging	Decreased (ΔΔ*G* = −0.84) Decreased (ΔΔ*G* = −0.49)	0	0	0
CCF02153	Chr. 2: 179650807	14	c.2138G>A	p.R713Q	Z-disk	Damaging	Decreased (ΔΔ*G* = −0.65)	0	0	2/120504 (1.66e-05), 0 hom
CCF06523	Chr. 2: 179403402	354	c.99154C>T	p.R33052C	A-band	Damaging	Decreased (ΔΔ*G* = −1.00)	0	0	5/120752 (4.141e-05), 0 hom
CCF02289	Chr. 2: 179396361	358	c.104981G>A	p.S34994N	M-band	Damaging	Decreased (ΔΔ*G* = −1.41)	0	0	0
CCF08133	Chr. 2: 179430434	305	c.80425G>A	p.G26809S	A-band	Damaging	Decreased (ΔΔ*G* = −0.67)	*T* = 1/C = 12369 (0.000081)	0	26/120702 (0.0002154), 0 hom

^a^ Genomic positions are according to the human reference haploid genome sequence, hg19 and variants reported corresponding to *TTN* transcript NM_001267550

^b^ Predicted through a combination of SIFT, MutationTaster, and PolyPhen-2 (see Materials and Methods)

^c^ Predicted through I-Mutant 2.0 program using the difference in the Gibbs free energy values, ΔΔ*G* = Δ*G* (mutant protein)—Δ*G* (Wildtype protein) in Kcal/mole. The sign of ΔΔ*G* predicts protein stability

^d^ Allele frequency data was extracted from the National Heart, Lung, and Blood Institute (NHLBI) Exome Sequencing Project (ESP) Exome Variant Server (http://evs.gs.washington.edu/EVS/) v.0.0.28, 1000 Genomes Project (http://www.internationalgenome.org), and Exome Aggregation Consortium (ExAC), Cambridge, MA (http://exac.broadinstitute.org) all last accessed August 10, 2017

^e^ ExAC note: “This variant is only covered in 27,830 individuals (adjusted allele number = 55660). This means that the site is covered in fewer than 80% of the individuals in ExAC, which may indicate a low-quality site”

**Fig. 1:**
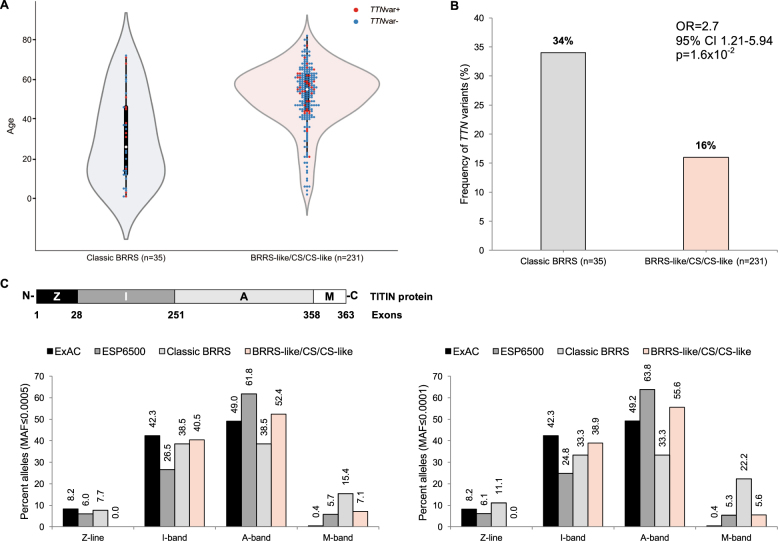
Characteristics of *TTN* variants in BRRS and population controls. **a** Distribution of ages at consent in years between classic BRRS (*n* = 35) and BRRS-like/CS/CS-like individuals (*n* = 231). Each colored dot represents an individual patient, with red dots indicating individuals with identified *TTN* variants (*TTNvar+*) and blue dots indicating those who are *TTN*-wildtype (*TTNvar*-). The central white dot indicates the median of the age distribution. **b** Frequency of *TTN* variants identified in classic BRRS (*n* = 35) and BRRS-like/CS/CS-like individuals (*n* = 231). **c** The four distinct regions of the TITIN protein (Z-disk, Z; I-band, I; A-band, A; M-band, M) are depicted with the corresponding encoding exon boundaries. N and C refer to the amino terminus and carboxy terminus of the protein, respectively. Graphs show the frequency of alleles in percent observed in our patient series and population controls at minor allele frequencies (MAF) cut-offs of 0.0005 (0.05%) and 0.0001 (0.01%). BRRS Bannayan–Riley–Ruvalcaba syndrome, CS Cowden syndrome

We next sought to examine whether *TTN* variants also exist in *PTEN-*wildtype individuals with Cowden syndrome (CS), CS-like meeting diagnostic criteria minus one criterion,^11,23^ or in individuals with BRRS-like features. We included 231 eligible patients in this analysis. The majority of these patients were female (*n* = 202 or 87%), which contrasts with the male-predominant (*n* = 31/35 or 89%) classic BRRS series (*p* < 10^-7^). Moreover, the median age of these patients was older at 55 ± 15 years (range: 2–82) compared to 26 ± 22 years (range: 1–72) for the classic BRRS patients (*p* = 2.0 × 10^-6^) (Fig. 1a). Through a combination of exome sequencing and *TTN*-targeted panel sequencing, we identified an additional 37 (16%) patients with *TTN* variants (Supplementary Table 5). We did not observe differences in age distribution between individuals who harbor a *TTN* variant versus those who are wildtype, whether in the classic BRRS (*p* = 3.1 × 10^-1^) or the BRRS-like and CS/CS-like (*p* = 1.9 × 10^-1^) series of patients (Fig. 1a). The frequency of *TTN* variants in classic BRRS patients remains notably higher compared to BRRS-like and CS/CS-like individuals (OR = 2.7, 95% CI 1.21-5.94, *p* = 1.6 × 10^-2^) (Fig. 1b). We further conducted an independent analysis of exomes sequenced for patients who do not have BRRS, serving as an additional internal control. Clinical phenotypes included sporadic polyposis (*n* = 11), Marfan syndrome and aortic diseases (*n* = 13), and epilepsy (*n* = 21). Using identical filtration and prioritization criteria as we have done for our BRRS patient exomes, we identified a total of 6/45 (13%) patients with *TTN* variants (Supplementary Table 6). The frequency we identified in classic BRRS (12/35 or 34%) still reflects enrichment compared to this unrelated data set with similar sequencing parameters (OR = 3.3, 95% CI 1.11–10.84, *p* = 3.2 × 10^-2^). Of note, this frequency in the internal control exomes might also be over-estimated since we included individuals who have certain overlapping phenotypes with BRRS (polyposis) and individuals who suffer from heart-related disease. Additionally, none of these variants were validated through Sanger sequencing, although we quality controlled them using the Integrated Genomics Viewer (IGV) browser.

### Comparative analysis of *TTN-*variant frequencies and spectra between patients and population controls

The enrichment of *TTN* variants in our patient series prompted us to agnostically analyze the frequencies of *TTN* missense variants within population controls, namely 1000G, NHLBI-ESP6500, and ExAC. Using similar variant prioritization criteria, we observe a mean variant frequency of 10% for all the three data sets at a MAF cut-off of 0.0005, and 4% at a MAF cut-off of 0.0001 (Table 3). From these databases, while we can determine the total number of alleles with variants, it is not possible to determine whether some of these alleles exist within the same individual (i.e., the absolute number of individuals harboring *TTN* variants). Considering only rare alleles, likely representing private variants observed in singleton exomes (MAF ≤ 0.0001), we note significant enrichment of *TTN* variants in our classic BRRS patient series compared to all 3 data sets collectively (34 versus 4% respectively, OR = 2.2, 95% CI 1.01-4.20, *p* = 4.7 × 10^-2^).

**Table 3:** Odds ratios (OR) of *TTN* germline variants in population controls compared to classic BRRS

MAF cut-off	Database	Number of alleles^a^	% of alleles	OR	95% CI	*P*-value
MAF ≤ 0.0005	1000G	231	4.6	4.276	2.176-7.891	0.0001189
	ESP6500	1895	14.6	1.213	0.624-2.209	0.5327
	ExAC	11811	11.1	1.654	0.8519-3.006	0.1281
MAF ≤ 0.0001	1000G	0	0	∞	∞	<0.0000001
	ESP6500	820	6.3	2.192	1.022–4.278	0.04441
	ExAC	6651	6.3	2.209	1.033–4.294	0.04202

^a^Allele frequency data was extracted from the National Heart, Lung, and Blood Institute (NHLBI) Exome Sequencing Project (ESP) Exome Variant Server (http://evs.gs.washington.edu/EVS/) v.0.0.28, 1000 Genomes Project (http://www.internationalgenome.org), and Exome Aggregation Consortium (ExAC), Cambridge, MA (http://exac.broadinstitute.org) all last accessed August 17, 2017

*MAF* minor allele frequency, *OR* odds ratio, *CI* confidence interval, ∞ approaching infinity

The TITIN protein is organized into four regions that correlate with the structurally and functionally distinct regions of the muscle sarcomere.^24^ These regions, from the amino terminus to the carboxy terminus of the protein, include the Z-disk, I-band, A-band, and the M-line. Since these different regions of TITIN show distinct patterns of expression and germline mutations in different disease contexts,^24,25^ we next examined whether the spectrum of variants we identified in our patients are distinct from those observed in the “control” data sets. Indeed, looking at the population controls (ExAC and ESP6500), we observe a trend towards increased number of variants (at both MAF ≤ 0.0005 and 0.0001) in the A-band versus the I-band (Fig. 1c). We observe a similar trend of A-band enrichment in BRRS-like/CS/CS-like individuals. In contrast, the classic BRRS patient series revealed equal distribution of variants between the A-band and the I-band. While the frequency of variants in the region encoding the Z-line in BRRS (7.7%) falls in between the variant frequencies from ExAC (8.2%) and ESP6500 (6%) at MAF ≤ 0.0005, we observe increased Z-line variant frequency in BRRS (11.1%) compared to population controls (ExAC, 8.2% and ESP6500, 6.1%) at MAF ≤ 0.0001. Notably, we also observe significant enrichment of variants in the M-band in classic BRRS compared to population controls and BRRS-like/CS/CS-like individuals, and this held true at both MAF cut-offs and more significantly at MAF ≤ 0.0001 (*p* = 2.1 × 10^-2^ at MAF ≤ 0.0005 and *p* = 6.7 × 10^-3^ at MAF ≤ 0.0001).

### Functional interrogation of *TTN* c.15286T>C, p.C5096R reveals lack of contact inhibition phenotype

**Fig. 2:**
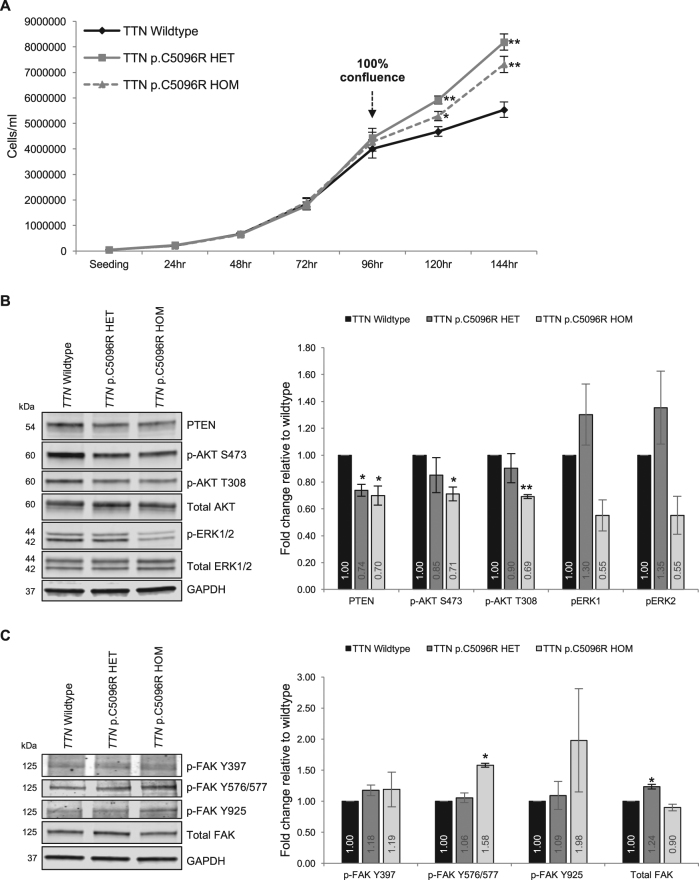
Functional interrogation of *TTN* c.15286T>C, p.C5096R HEK293T cells reveals lack of contact inhibition phenotype. **a** Wildtype and mutant cells were serially counted at 24-h interval for up to 144 h after seeding. Trypan blue stain was used to count dead cells and assess viability. Experiments were performed as four biological replicates, and counted at least in triplicates at each time point. Data represent pooled mean values ± SEM. **p < 0.001, *p < 0.05 (two-sided Student’s *t* test comparing each mutant cell line to wildtype). **b** Western blot and densitometric analysis of some downstream readouts of the PTEN signaling pathway. Blots were derived from the same experiment and representative of three independent biological replicates, with protein samples from each cell line (genotype) processed in parallel within each experiment. Densitometric data represent pooled mean values of the replicate experiments ± SEM. **p < 0.005, *p < 0.05 (two-sided Student’s *t* test comparing each mutant cell line to wildtype). **c** Western blot and densitometric analysis of total and phosphorylated FAK protein. Blots were derived from the same experiment and representative of two biological replicates, with protein samples from each cell line (genotype) processed in parallel within each experiment. p-FAK Y397, autophosphorylation site at tyrosine residue 397; p-FAK Y576/577, catalytic domain phosphorylation at tyrosine residues 576/577; p-FAK Y925, carboxy-terminal region phosphorylation at tyrosine residue 925. Densitometric data represent pooled mean values of the replicate experiments ± SEM. *p < 0.05 (two-sided Student’s *t* test comparing each mutant cell line to wildtype). Both Western blot experiments were performed on lysates from cells harvested at the 144 h timepoint (Day 6). Non-phosphorylated proteins are normalized to GAPDH (loading control), whereas phosphorylated proteins are normalized to corresponding total protein levels after normalization of both proteins to GAPDH (ratio of the ratios). Fold changes are calculated relative to wildtype (set to 1). Molecular weights of proteins in kilo Daltons (kDa) are indicated on the left side of each blot

The functional consequences of *TTN* variants have been extensively evaluated in the context of dilated cardiomyopathy (DCM).^25–29^ In this context, one study reported the association of a *TTN* truncating variant with decreased growth factor signaling pathway readouts, such as diminished phosphorylation of MAPK and AKT,^28^ which are pertinently downstream of PTEN signaling. Relevant to BRRS, we hypothesized that the *TTN* variants we observe could result in downstream cellular phenotypes and signaling readouts similar to PTEN loss of function (i.e., increased growth and activation of the MAPK and/or AKT pathways). We utilized CRISPR/Cas9 genome-edited HEK293T cells that are wildtype for *TTN*, or harboring one (heterozygous, *TTN* p.C5096R HET) or two (homozygous, *TTN* p.C5096R HOM) mutant alleles of a variant we observed in a classic BRRS patient (c.15286T>C, p.C5096R; CCF01021) (Supplementary Figs. 1, 2). We include the homozygous mutant cells since we have observed some patients with more than one variant. We did not observe differences in growth between the three genotypes up until 96 h after seeding. Intriguingly, when the cells reach 100% confluence, the differences become apparent, with both mutant cell lines growing faster than the wildtype and forming foci of cells growing over each other (Fig. 2a and Supplementary Fig. 3). These differences were independent of cell viability and migration (Supplementary Fig. 4) and suggested lack of contact inhibition in mutant cells.

We next investigated the PTEN pathway through Western blot analysis. Interestingly, while we observe a decrease in PTEN expression in mutant cells compared to wildtype cells, we note paradoxical downstream readouts of decreased phosphorylation of AKT and ERK1/2, the latter trends more prominent in the homozygous mutant cells (Fig. 2b). Relevant to the contact inhibition phenotype downstream of PTEN signaling,^30^ we then assayed FAK expression and phosphorylation status and indeed noted increased levels of total FAK in *TTN* p.C5096R HET mutant cells and increased p-FAK (Y576/577) in *TTN* p.C5096R HOM mutant cells compared to wildtype (Fig. 2c).

## Discussion

Our observations reveal that a subset of classic BRRS individuals without germline *PTEN* mutations might be accounted for by germline variants in *TTN*. The *TTN* gene encodes TITIN, also known as Connectin, an abundant protein with key structural and functional roles in vertebrate striated muscles.^31^ TITIN is the largest known human protein (~34,000 amino acids with molecular weight of ~3.8 MDa) encoded by a giant gene (363 exons for the longest transcript).^32^ Germline *TTN* mutations have been linked to a multitude of cardiac and/or skeletal muscle Titinopathies (Supplementary Figs. 5–7).^25^ Interestingly, neuromuscular phenotypes, such as joint hyperextensibility, muscle hypotonia, lipid storage myopathy, and delayed psychomotor development, have been observed in BRRS patients.^1,2,4^ Muscle weakness and hypotonia cause affected BRRS infants to appear “floppy” and with delayed development of motor skills. None of our BRRS patients had a reported personal or family history of heart disease, and we speculate that the type and spectrum of the variants observed and their existence in particular protein domains may contribute to the observed clinical phenotypes in these distinct disorders. A comprehensive modeling of this concept has been recently investigated in DCM.^27^ *TTN* truncating mutations (*TTN*tv) were described in ~25% of familial and 18% of sporadic cases of DCM, with enrichment of mutations in the A-band of TITIN.^29^ A more recent study showed that exons that are constitutively expressed in the heart are significantly associated with DCM and can occur in all TITIN protein domains.^27^ Importantly, distal I-band and all A-band *TTN*tv resulted in the highest odds ratios (ORs), suggesting position-dependent effects that modify DCM penetrance.^27^ In contrast, all our germline *TTN* variants in classic BRRS are missense and are equally distributed between the I-band and A-band, being particularly enriched in the M-band of the protein. Importantly, the M-band contains a constitutively expressed TITIN kinase (TK) domain, encoded by human *TTN* exon 358 (amino acids 33819-34073), making TITIN a member of the Ca^2+^/calmodulin-dependent serine/threonine protein kinase family.^33^ *TTN* M-line homozygous or compound heterozygous mutations have been associated with multiminicore disease (MmD) with pediatric heart disease,^34^ and findings from this and other studies suggest that the M-line region is particularly important for maintenance of skeletal muscle integrity.^35^ We identified *TTN* exon 358 M-band variants in 2/12 (17%) classic BRRS and 3/37 (8%) BRRS-like individuals with identified *TTN* variants. All variants are downstream of the TK protein domain. Although based on a small sample size, it is tempting to speculate whether such enrichment is uniquely pertinent to classic BRRS, and hence, warrants further investigation.

We also found that rare (MAF ≤ 0.0001) germline variants were enriched in our classic BRRS patient series compared to BRRS-like and CS/CS-like individuals (OR = 2.7, 95% CI 1.21–5.94, *p* = 1.6 × 10^-2^) and multiple population controls (OR = 2.2, 95% CI 1.009-4.195, *p* = 4.7 × 10^-2^). This enrichment was only apparent compared to the 1000G data set at MAF ≤ 0.0005. This could be explained either due to the presence of individuals with heart-related diseases within the NHLBI-ESP6500 and ExAC data sets, or due to the smaller sampled allele number in 1000G (*n* = 5008) compared to the former two data sets (*n* = 13006 and *n* = 106210, respectively). Despite these subtle differences in *TTN* variant frequencies at MAF ≤ 0.0005 between classic BRRS and population controls, it is important to note that the variant distribution remained consistent, with M-band variant enrichment and equal distribution of variants between the A-band and the I-band. Therefore, it is also possible that the spectrum of *TTN* variants in different functional protein domains could be contributing to BRRS-relevant phenotypes compared to population controls. This is particularly intriguing since *TTN* undergoes extensive tissue-specific splicing, with preferential expression of certain exons within particular tissues.^26^ It is also worth mentioning that *TTN*tv have been observed in ~1% of the general population in the absence of an apparent DCM phenotype, which prompted extensive phenotyping of such individuals to then confirm eccentric cardiac geometry and abnormalities in heart function in these apparently healthy individuals.^27^ Therefore, whether such individuals with extremely rare *TTN* variants existing in population controls have BRRS or BRRS-like features remains unclear. The rarity of BRRS as a disorder also makes expanding our analyses to determine more conclusive genotype–phenotype associations more difficult.

BRRS individuals harboring germline *PTEN* mutations belong to the PHTS, which also includes that subset of Cowden, Cowden-like, Proteus, and Proteus-like syndromes characterized by germline *PTEN* mutations.^15^ As a tumor suppressor, a defective PTEN protein or PTEN haploinsufficiency (e.g., from decreased expression) can lead to increased cell growth and proliferation (size and/or number), explaining the presence of hamartomas (from Greek *hamartion* meaning a bodily defect consisting of benign and disorganized overgrowths of resident cells) and other overgrowths in *PTEN* mutation positive patients.^6,15^ Although TITIN has been shown to be an extensive signaling node in muscle tissue,^36^ little is known about muscle-independent functions. TITIN protein isoforms have been reported in smooth muscles and non-muscle cells such as fibroblasts and platelets as a component of cellular stress fibers.^37^ Intriguingly, other reports suggest that non-muscle TITIN could exist in the nucleus, interacting with histone proteins,^38^ further supporting a role as a chromosome scaffold^39^ and a factor important for maintaining genome integrity. These observations support a possible role of TITIN in muscle-independent contexts, and particularly as relevant to overgrowth phenotypes.

Our functional data, though limited to one variant, suggest that TITIN could play a role in the PTEN signaling pathway. The reason behind decreased PTEN protein expression in mutant cells and whether TITIN is directly responsible for this change in expression remains unclear. Interestingly, we observe a similar reduction in canonical PTEN downstream signaling readouts (AKT and ERK) as was observed in DCM-*TTN*tv-related studies.^28^ Although this was surprising and contrary to our expectations given the decreased level of PTEN and in the context of an overgrowth syndrome, we suspect that the cells activate negative feedback loops to circumvent the contact inhibition. However, this does not explain whether decreased PTEN expression is directly affected by the *TTN* genotype or by another feedback response to alleviate the decreased activation of AKT and/or ERK. Therefore, the precise mechanism of how these observations happen (e.g., dampening PI3K or other growth factor signaling or activating other tumor suppressors regulating AKT/MAPK pathway) certainly warrant further investigation. Our data show that for the particular variant we studied, contact inhibition of proliferation could be the determinant of the observed overgrowth phenotypes. This could be mediated by the increased FAK expression or phosphorylation at its catalytic residue (Y576/577, Fig. 2c), the former known to be regulated by PTEN^30^ and to regulate contact inhibition itself.^40,41^ Interestingly, PTEN has also been shown to be phosphorylated by FAK at Tyr336, downstream of RhoA/ROCK, resulting in association of PTEN to the membrane, increased PTEN phosphatase activity, and protein stability.^42^ In other contexts, FAK phosphorylation of PTEN causes PTEN to enter the nucleus (and hence, resulting in increased degradation). As PTEN protein stability is regulated through ubiquitination, and mono-ubiquitination of PTEN is crucial for nuclear import,^43^ we predict that the cross-talk between FAK and PTEN could also regulate PTEN subcellular localization and overall stability. Therefore, it seems that PTEN-FAK signaling represents a context-dependent push–pull axis, whereby FAK can promote oncogenic signaling pathways and yet activate PTEN growth-suppressive signaling in particular contexts. In the context of *TTN* variants and given our experimental observations, we suspect that TITIN could function as a sensor upstream of FAK, sustaining growth-promoting signals even in the existence of contact inhibition. Indeed, an interesting observation comes from the BioGRID database.^44^ In looking at curated TITIN-interacting partners, we observe several pertinent proteins (Supplementary Fig. 8). As is observed, it is possible that TITIN interacts with PTEN pathway-relevant proteins, including epidermal growth factor receptor, mitogen-activated protein kinase 1 (MAPK1 or ERK2), and E-cadherin (CDH1). This indeed adds even more complexity to the biology of *TTN* variants and their possible roles in modulating the push–pull hypothesis of context-dependent proliferation versus growth inhibition. We also find the possible association of TITIN with cellular structural and cytoskeletal components such as CDH1 and ACTN1 as intriguing, since TITIN has been reported to exist as a component of cellular stress fibers,^37^ also important for sensing the microenvironment and regulating cell migration and growth (also as relevant to FAK).

**Fig. 3:**
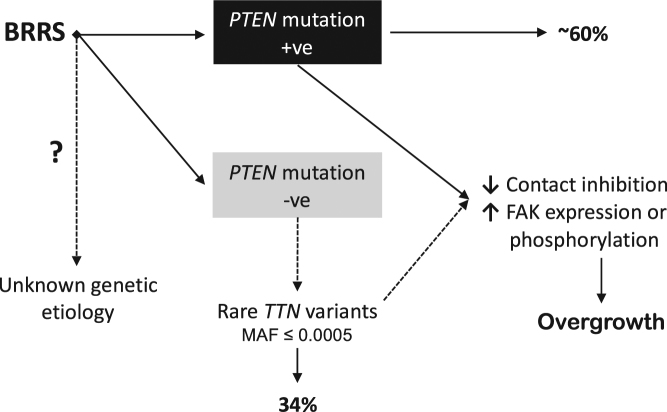
Germline *TTN* variants are enriched in *PTEN-*wildtype BRRS. BRRS Bannayan-Riley-Ruvalcaba syndrome, +ve positive, -ve negative, MAF minor allele frequency

Our findings do not exclude the possibility that genes other than *TTN* could be playing a role in *PTEN-*wildtype BRRS. Throughout our exploratory analysis of this classic BRRS series (*n* = 35), we identified other genes with variants in three or more individuals. These genes were not as enriched as *TTN* (*n* = 12) and include *AK9* (*n* = 4), *ANKAR* (*n* = 3), *CDH24* (*n* = 3), *ITPR3* (*n* = 3), *SSPO* (*n* = 3), and *STARD9* (*n* = 3). Interestingly, TITIN interacts with adenylate kinase (AK) proteins,^36^ and the Inositol 1,4,5-Trisphosphate Receptor Type 3 encoded by *ITPR3* is regulated by PTEN.^45^ Relatedly, using the same filtration criteria implemented to prioritize *TTN*, we did not find any variants in other genes known to be associated with CS, namely *SDHx*, *AKT1*, *PIK3CA*, *SEC23B*, and *USF3*,^46–49^ in our patients with germline *TTN* variants. Overall, our findings that germline missense *TTN* variants are enriched in individuals with classic BRRS compared to BRRS-like individuals and population controls are supported by our exploratory functional data to suggest a possible role for TITIN in overgrowth phenotypes. Importantly, these findings provide a helpful molecular diagnostic marker as BRRS potentially joins the growing list of Titinopathies (Fig. 3).

## Methods

### Research participants and clinical data

Research participants were prospectively (2011-2015) accrued broadly from both community and academic medical centers throughout North America, Europe (>85% originating from these two continents) and Asia using a standard protocol.^50^ Eligible probands were BRRS patients with classic clinical features (typically showing the triad of macrocephaly, lipomatosis, and pigmented macules of the glans penis in males), and who tested negative for germline mutations in *PTEN* (single nucleotide variations and large deletions). Scanning of genomic DNA for *PTEN* mutations (including promoter region) was performed as we previously reported.^46,51^ Deletion analysis was performed for *PTEN* using the multiplex ligation-dependent probe amplification assay, according to manufacturer’s protocol.^52^ For the targeted mutation analysis of the expanded patient series, eligible patients met at least the relaxed International Cowden Consortium operational diagnostic criteria (Supplementary Table 7). Relaxed criteria are defined as full criteria minus one and such individuals are referred to as CS-like.^23^ These patients were also enriched for phenotypic features overlapping with BRRS, including macrocephaly, lipomas, hemangiomas, vascular malformations, intestinal polyposis, autism/developmental delay, and thyroid involvement (Hashimoto’s thyroiditis or nodules). Medical records were reviewed for each patient and family history extracted from clinical genetics and genetic counseling visit notes, where applicable, and with the patients’ consent. The methods were performed in accordance with relevant guidelines and regulations and approved by the Cleveland Clinic Institutional Review Board (IRB protocol #8458). Informed consents from all research participants were obtained for this study.

### Exome sequencing and variant filtration

We subjected germline genomic DNA extracted from peripheral blood leukocytes to paired-end exome sequencing using Illumina HiSeq 2500 platform. Sequencing was performed at three independent sites: Genomics Core of the Lerner Research Institute of the Cleveland Clinic (Cleveland, OH), Personalis Genome Services (Menlo Park, CA) implementing the ACE Exome™ technology for optimal gene targeting and coverage, and the Broad Institute’s Genomics Platform (Cambridge, MA). To prioritize causal variants, we applied the Annovar Variants Reduction pipeline^53^ and as previously reported.^48^ All resultant variants were manually inspected through the IGV^54,55^ and validated using PCR-based region-specific mutation analysis through Sanger sequencing using 3730xl DNA Analyzers at the Genomics Core of the Lerner Research Institute of the Cleveland Clinic (Cleveland, OH). Resultant chromatograms were analyzed using the Mutation Surveyor DNA Variant Analysis Software (SoftGenetics, State College, PA) and variants reported according to the Human Genome Variation Society and subsequently cross-matched with the exome findings.

### Targeted sequencing of *TTN*

We used the TruSight Cardiomyopathy sequencing panel (Illumina, San Diego, CA), covering 46 cardiomyopathy target genes, including *TTN*. Sequencing was done using Illumina HiSeq 2500 platform, rapid run, 100 bp paired-end reads and at 150× coverage per sample. We also designed 13 primer pairs to cover 20 exons within the I-band (*TTN* transcript NM_001267550) that are not covered by the TruSight panel. The specific exons, primer sequences, and PCR cycling conditions are listed in Supplementary Table 8. Resultant amplicons were then subjected to Sanger sequencing as mentioned above.

### Interpretation of *TTN* variants for prioritization and downstream analysis

Variants retained after filtration were further evaluated in silico using a combination of mutation-prediction algorithms, namely SIFT,^20^ MutationTaster,^56^ PolyPhen-2,^57^ and I-Mutant.^22^ Variants were retained if they were predicted to be damaging according to SIFT, MutationTaster, and PolyPhen-2 HVAR and/or HDIV. Variants that have been previously reported were inspected manually through the 1000 Genomes Project server, the National Heart, Lung, and Blood Institute (NHLBI) Exome Sequencing Project (ESP) Exome Variant Server (ESP6500SI-V2), and the Exome Aggregation Consortium (ExAC) browser, to identify the minor allele frequencies.

### Analysis of *TTN* variants from patient and population controls

We analyzed the frequency and spectrum of *TTN* variants from the 1000 Genomes Project (1000G, August 2015 release), the NHLBI ESP Exome Variant Server (ESP6500 data set), and the Exome Aggregation Consortium (ExAC) excluding The Cancer Genome Atlas (TCGA) data set. We analyzed variants according to 2 (MAF) cut-offs: (1) 0.0005 or 0.05% representing the MAF cut-off used in the Annovar variant prioritization pipeline for gene discovery, and (2) 0.0001 or 0.01% representing more rare variants. We further conducted an independent analysis of exomes sequenced for patients who do not have BRRS, with some sequenced at the same time as our BRRS patient exomes, serving as an additional internal control. These included sporadic polyposis (*n* = 11), Marfan syndrome and aortic diseases (*n* = 13), and epilepsy (*n* = 21) patient series. *TTN* transcript variant IC (NM_001267550) was used for all analyses. We prioritized exonic missense variants and utilized the same filtration criteria used to prioritize *TTN* variants from patient exomes.

### Cell lines and culture conditions

HEK293T CRISPR/Cas9 genome-edited knock-in stable cell lines were generated using the cell-based assay screening service of Baylor College of Medicine (Houston, TX). Individual clones harboring three different genotypes were isolated and verified: (1) *TTN* Wildtype, (2) *TTN* p.C5096R heterozygous, and (3) *TTN* p.C5096R homozygous. We generated wildtype and heterozygous pools of cells, each consisting of 3 clones of cells. The *TTN* genotype at the locus of interest (c.15286T>C, p.C5096R) was validated using Sanger sequencing (Supplementary Fig. 2). The HEK293T cells were cultured in DMEM supplemented with 10% fetal bovine serum and 1% penicillin and streptomycin. Cell lines were maintained at 37 °C and 5% CO_2_ culture conditions and tested negative upon routine mycoplasma testing with the MycoAlert Mycoplasma Detection Kit (Lonza) at the C.E. lab (luminescence ratios < 0.9) prior to downstream functional interrogation.

### Doubling time and cell viability

Cells were seeded in 24-well plates at a density of 25,000 cells per well and allowed to grow overnight. At each timepoint, cells were trypsinized, homogenized, and counted using the Countess automated cell counter (Invitrogen, Waltham, MA). We used Trypan blue to account for dead cells and assess cell viability. Counting experiments for all genotypes were run in parallel. All counts were done at least in three technical replicates, and counting was done in four biological replicates.

### Immunoblotting

Protein was extracted from whole cell lysates using the Mammalian Protein Extraction Reagent M-PER (Thermo Scientific Pierce, Rockford, IL) supplemented with a cocktail of protease and phosphatase inhibitors (Sigma-Aldrich, St. Louis, MO) and quantified through the BCA protein assay (Thermo Scientific Pierce). Lysates were separated by SDS-PAGE and transferred onto nitrocellulose membranes. We probed for anti-PTEN (Cascade Bioscience clone 6H2.1) at 1:2000, anti-phospho-AKT S473 (Cell Signaling #4060) at 1:1000, anti-phospho-AKT T308 (Cell Signaling #2965) at 1:1000, anti-total AKT (Cell Signaling #9272) at 1:1000, anti-phospho-ERK1/2 (Cell Signaling #9101) at 1:1000, anti-ERK1/2 (Cell Signaling #9102) at 1:1000, anti-phospho-FAK Y397 (Cell Signaling #8556) at 1:1000, anti-phospho-FAK Y576/577 (Cell Signaling #3281) at 1:1000, anti-phospho-FAK Y925 (Cell Signaling #3284) at 1:1000, anti-FAK (Cell Signaling #13009) at 1:1000, and anti-GAPDH (Cell Signaling #2118) at 1:40,000 dilutions. For each experiment, all genotypes were run in parallel on the same gel. Blots were scanned digitally and quantified using the Odyssey Infrared Imaging System (Li-Cor Biosciences, Lincoln, NE) and Image J software (NIH, Bethesda, MD). Original blot images are shown in Supplementary Fig. 9.

### Statistical analysis

Statistical analyses were conducted using OpenEpi software (Open Source Statistics for Public Health, http://www.openepi.com/). For analyses between affected population groups, we used 2 × 2 tables to calculate the OR and 95% CI and used the mid-p exact test to calculate corresponding *p* values. Experimental data between wildtype and mutant cell lines are given as means ± SEM. Student’s *t* test was used for significance testing as indicated in the figure legends. All statistical tests were two sided, and *p* values ≤ 0.05 were deemed significant.

### Web resources

Annotate Variation, Annovar: http://www.openbioinformatics.org/annovar/

Exome Aggregation Consortium, ExAC: http://exac.broadinstitute.org/

Genome Analysis Toolkit, GATK: http://www.broadinstitute.org/gatk/

I-Mutant 2.0: http://folding.biofold.org/i-mutant/i-mutant2.0.html

Integrative Genomics Viewer, IGV: https://www.broadinstitute.org/igv/home

MutationTaster: http://www.mutationtaster.org/

NHLBI-ESP Exome Variant Server, NHLBI-ESP6500: http://evs.gs.washington.edu/EVS/

Polymorphism Phenotyping v2, PolyPhen-2: http://genetics.bwh.harvard.edu/pph2/

Sorting Intolerant from Tolerant, SIFT: http://sift.jcvi.org/

1000 Genomes Project, 1000G: http://www.internationalgenome.org

### Accession numbers

GenBank accession numbers are included in the Materials and Methods section and as a footnote in tables listing gene variants.

### Data availability

All analyses are included in the figures, tables, and supplementary information files.

## Electronic supplementary material

Supplemental Material
