# Supplementary material for: Germline TTN variants are enriched in PTEN-wildtype Bannayan–Riley–Ruvalcaba syndrome
Source: NPJ Genom Med. 2017 Dec 18;2:37. doi: 10.1038/s41525-017-0039-y (PMC5735137; doi:10.1038/s41525-017-0039-y)
Supplement: Supplementary file 1 — Supplemental Material [file 41525_2017_39_MOESM1_ESM.pdf]

Supplementary Appendix

Germline *TTN* Variants are Enriched in *PTEN*-Wildtype Bannayan-Riley-Ruvalcaba Syndrome

Lamis Yehia, Ying Ni, and Charis Eng

SUPPLEMENTARY METHODS

In vitro scratch migration assay

Cells were grown overnight and migration assay performed according to Liang et al.,[1] with the minor variation of creating scratches on the cell monolayer using a p10 pipet tip. We measured migration distance after 24 hours. Cells were photographed under 10x magnification and distances measured in triplicate fields of view. Images were taken using a Leica DMI3000B manual inverted microscope (Leica, Buffalo Grove, IL).

Reference

1. Liang, C.C., Park, A.Y. & Guan, J.L. 2007. In vitro scratch assay: a convenient and inexpensive method for analysis of cell migration in vitro. *Nat Protoc* **2**:329-33. 10.1038/nprot.2007.30.

Supplementary Table 1. Clinical and demographic characteristics of 14 unrelated probands with classic BRRS features

Subject	Age at consent	Gender	Macrocephaly	Penile freckling	Neuro-psychological	Benign overgrowths and skin features	Other phenotypes and incidental findings
CCF00162	33	M	Yes (59 cm)	Yes	Unknown	Lipoma, hemangioma (NOS)	Prominent Schwalbe's lines
CCF02011	1	M	Yes (59 cm)	Yes	Developmental delay/ASD	Unknown	Unknown
CCF06949	51	M	Yes (58.5 cm)	Yes	Developmental delay/ASD	Skin hemangioma	Obsessive compulsive disorder
CCF07265	68	M	Yes (61 cm)	Yes	Unknown	Mucocutaneous lip pigmentation	Prostate cancer (age 65)
CCF06673	15	M	No	Yes	Unknown	Skin fibroma	Follicular variant papillary thyroid cancer (age 13)
CCF00155	24	M	No	Yes	Developmental delay/ASD	Benign thyroid nodule, papillomatous papules (NOS), arteriovenous malformation	Unknown
CCF04693	12	M	No	Yes	Developmental delay/ASD	Juvenile polyp (n=1), intestinal polyposis (>10)	Café-au-lait macules, inguinal freckling, multiple naevi, hyperflexibility
CCF07445	38	M	Yes (59.2 cm)	Yes	Unknown	Trichilemmoma, acral keratoses, skin tag	Unknown
CCF01021	31	M	Yes (63.5 cm)	Yes	Mental retardation	Skin hemangioma, papillomatous papules (mucosa)	Hypotonia, hydrocephalus
CCF02423	45	M	Yes (59 cm)	Yes	Developmental delay/ASD	Unknown	Melanosis coli, melanoma in situ (age 41), renal clear cell cancer (age 42)
CCF05285	46	M	Yes (59 cm)	Yes	Unknown	Skin fibroma, acral keratoses, papillomatous papules, polyps	Thyroid cancer (age 23)
CCF06892	53	M	Yes (59 cm)	Yes	Unknown	Trichilemmoma, acral keratoses, skin hemangioma, polyps	Unknown
CCF08441	59	M	No	Yes	Unknown	Lipoma, skin fibroma, skin, vascular, and spinal hemangiomas, thyroid nodule	Melanosis coli, hearing loss, trigeminal neuralgia
CCF06480	23	M	Yes (62.3 cm)	Yes	Developmental delay/ASD	Unknown	Unknown

Abbreviations: M, male; cm, centimeters; NOS, not otherwise specified.

Supplementary Table 2. Shared genes with germline variants in at least 2 classic BRRS patients (n=14)

Gene	Description	Patients	Variants	MutationTaster
TTN	Titin	CCF00162	c.66187G>C, p.V22063L	Disease-causing
		CCF02011	c.33856G>A, p.E11286K	Disease-causing
		CCF06949	c.28549G>A, p.V9517M	Disease-causing
		CCF07265	c.92488G>A, p.V30830I	Disease-causing
		CCF06673	c.23497G>C, p.G7833R	Disease-causing
		CCF01021	c.15286T>C, p.C5096R	Disease-causing
		CCF02423	c.29317G>A, p.A9773T	Disease-causing
		CCF07445	c.68770G>A, p.A22924T	Disease-causing
			c.104575C>T, p.R34859W	Disease-causing
TRAP1	TNF receptor-associated protein 1	CCF00155	c.512A>C, p.N171T	Disease-causing
		CCF00162	c.778G>T, p.D260Y	Disease-causing
		CCF04693	c.591C>G, p.I197M	Disease-causing
DNAH11	Dynein Axonemal Heavy Chain 11	CCF00162	c.4367C>T, p.A1456V	Disease-causing
		CCF08441	c.11249C>T, p.A3750V	Disease-causing
FRAS1	Fraser Syndrome 1	CCF00162	c.7988C>T, p.A2663V	Disease-causing
		CCF07265	c.4273G>A, p.G1425R	Disease-causing
FRMD6	FERM Domain Containing 6	CCF05285	c.1187G>C, p.R396P	Disease-causing
		CCF06892	c.1763A>C, p.Q588P	Disease-causing
HEXA[a]	Hexosaminidase Subunit Alpha	CCF07265	c.805G>A, p.G269S	Disease-causing
		CCF02423	c.805G>A, p.G269S	Disease-causing
ITGA7	Integrin Subunit Alpha 7	CCF06892	c.473A>C, p.D158A	Disease-causing
		CCF02423	c.1345C>T, p.R449C	Disease-causing
LTA4H	Leukotriene A4 Hydrolase	CCF06673	c.164T>G, p.L55W	Disease-causing
		CCF02423	c.1096C>G, p.L366V	Disease-causing
PXDN	Peroxidasin	CCF04693	c.2021G>A, p.R674Q	Disease-causing
		CCF05285	c.2621G>A, p.R874H	Disease-causing
SEZ6L2	Seizure Related 6 Homolog Like 2	CCF06673	c.1855C>T, p.P619S	Disease-causing
		CCF08441	c.391G>A, p.A131T	Disease-causing
THBS2	Thrombospondin 2	CCF05285	c.3242C>T, p.A1081V	Disease-causing
		CCF04693	c.1105G>A, p.E369K	Disease-causing

[a]Known disease mutation (rs121907954, HGMD ID CM890061) for Tay-Sachs disease (TSD). TSD is autosomal recessive and both patients were heterozygous for the mutation.

Gene transcripts used to call variants: *TTN* (NM_001267550), *TRAP1* (NM_016292), *DNAH11* (NM_003777), *FRAS1* (NM_025074), *FRMD6* (NM_152330), *HEXA* (NM_000520), *ITGA7* (NM_001144997), *LTA4H* (NM_000895), *PXDN* (NM_012293), *SEZ6L2* (NM_201575), *THBS2* (NM_003247).

Supplementary Table 3. Clinical and demographic characteristics of 21 unrelated probands with classic BRRS

Subject	Age at consent	Gender	Macrocephaly	Penile freckling	Neuro-psychological	Other phenotypes
CCF02153	3	M	Yes (57.2 cm)	Yes	Developmental delay/ASD	Not observed
CCF06523	26	M	No (54 cm)	Yes	Unknown	Lipoma
CCF02289	46	M	No (55.5 cm)	Yes	Unknown	Lipoma
CCF00583	62	F	Yes	NA	Developmental delay/ASD	Thyroid goiter, fibrocystic breast disease, papillomatous papules, lipoma, uterine fibroids, renal cell cancer (age 62)
CCF00672	12	M	Yes	Yes	Developmental delay/ASD	Not observed
CCF03468	1	F	Yes (48 cm)	NA	Developmental delay/ASD	Lipoma, hemangioma, thyroid nodule, hypotonia
CCF04432	5	M	Yes (56 cm)	Yes	Developmental delay/ASD	Not observed
CCF04433	13	M	Yes (59 cm)	Yes	Developmental delay/ASD	Not observed
CCF04532	37	M	No	Yes	No	Lipoma, GI polyps, rectal cancer (age 26)
CCF04588	35	F	Yes (62 cm)	NA	Developmental delay/ASD	Lipoma, endometrial cancer (age 35)
CCF05173	20	M	Yes (61.2 cm)	Yes	Developmental delay/ASD	Not observed
CCF07875	5	M	Yes (56.4)	Yes	No	Not observed
CCF00006	60	M	Yes (63.2)	Yes	Unknown	Thyroid nodule, papillomatous papules, lipoma, fibroma, hemangioma, pectus excavatum
CCF00051	37	M	Yes (59.3)	Yes	No	Trichilemmoma, papillomatous papules, lipoma, GI polyps
CCF00241	16	M	Yes (59 cm)	Yes	No	Not observed
CCF00123	10	F	Yes	NA	Developmental delay/ASD	Lipoma
CCF01646	72	M	No (54.7 cm)	Yes	No	Papillary thyroid cancer (unknown age)
CCF02160	4	M	Yes	Yes	Developmental delay/ASD	Hemangioma
CCF03519	14	M	No (57.5)	Yes	Unknown	Not observed
CCF05149	14	M	Yes (58.5)	Yes	No	GI polyps
CCF08133	71	M	Yes (59.6)	Yes	Unknown	Follicular variant papillary thyroid cancer (age 68), goiter, benign breast disease, lipoma, visceral hemangioma, renal cell cancer (age 64)

Abbreviations: M, male; F, female; cm, centimeter; NA, not applicable; ASD, autism spectrum disorder; GI, gastrointestinal.

Supplementary Table 4. Shared genes with germline variants in at least 3 classic BRRS patients (n=35)

Gene	Description	Patients	Variants	MutationTaster
AK9	Adenylate Kinase 9	CCF02153	c.3895C>T, p.R1299W	Disease-causing
		CCF03468	c.4204C>T, p.R1402C	Disease-causing
		CCF06523	c.4814A>C, p.K1605T	Polymorphism
		CCF04693	c.3419G>T, p.G1140V	Disease-causing
ANKAR	Ankyrin And Armadillo Repeat Containing	CCF00241	c.3044T>C, p.V1015A	Disease-causing
		CCF05149	c.3058_3061delAAGG, p.K1020Tfs*22	Disease-causing
		CCF04433	c.4258C>T, p.L1420F	Polymorphism
CDH24	Cadherin 24	CCF04588	c.1387G>A, p.V463M	Disease-causing
		CCF00672	c.2264C>A, p.P755H	Polymorphism
		CCF05285	c.622G>T, p.V208L	Polymorphism
ITPR3	Inositol 1,4,5-Trisphosphate Receptor Type 3	CCF00051	c.4475A>T, p.Q1492L	Disease-causing
		CCF00672	c.6350A>G, p.Y2117C	Disease-causing
		CCF06673	c.309A>T, p.Q103H	Disease-causing
SSPO	SCO-Spondin	CCF00241	c.7586G>C, p.R2529P	ND
		CCF01646	c.8311+1G>A	ND
		CCF02153	c.6959C>T, p.T2320I	ND
STARD9	StAR Related Lipid Transfer Domain Containing 9	CCF03468	c.986G>A, p.R329Q	Polymorphism
		CCF02153	c.13808G>A, p.R4603Q	Disease-causing
		CCF02289	c.10034C>T, p. P3345L	Disease-causing

Gene transcripts used to call variants: *AK9* (NM_001145128), *ANKAR* (NM_144708), *CDH24* (NM_144985), *ITPR3* (NM_002224), *SSPO* (NM_198455), *STARD9* (NM_020759).

Abbreviations: ND, not determined. MutationTaster annotation problem (No start ATG exon found).

Supplementary Table 5. Exome sequencing and *TTN*-targeted sequencing identifies additional *TTN* germline variants in 37/231 (16%)

BRRS-like and CS/CS-like patients

Subject	Genomic position[a]	Exon	Variant	Protein alteration	Region	*In silico* predictions[b]	Protein stability[c]	NHLBI-ESP[d]	1000G[d]	ExAC[d]
CCF00627	Chr. 2: 179598073	54	c.15947C>T	p.A5316V	I-band	Damaging	Decreased (ΔΔG = 0.17)	0	0	0
CCF01065	Chr. 2: 179640730	28	c.5861C>A	p. P1954Q	I-band	Damaging	Decreased (ΔΔG = -0.42)	0	0	0
CCF01705	Chr. 2: 179571287	102	c.29314G>A	p.V9772M	I-band	Damaging	Decreased (ΔΔG = -0.33)	0	2/5008 (0.0004)	8/120748 (6.625e-05), 0 hom
CCF02331	Chr. 2: 179597669	55	c.16234G>T	p.A5412S	I-band	Damaging	Decreased (ΔΔG = -0.83)	0	0	0
CCF02413	Chr. 2: 179451362	308	c.64266A>C	p.K21422N	A-band	Damaging	Decreased (ΔΔG = -0.59)	0	0	0
CCF02462	Chr. 2: 179597782<br>Chr. 2: 179424184<br>Chr. 2: 179398570	55<br>326<br>358	c.16121G>A<br>c.86675G>C<br>c.102772C>A	p.C5374Y<br>p.W28892S<br>p.P34258T	I-band<br>A-band<br>M-band	Damaging	Decreased (ΔΔG = -1.13)<br>Decreased (ΔΔG = -2.41)<br>Decreased (ΔΔG = -1.56)	T=1/C=12165 (0.000082)<br>0<br>0	0<br>0<br>0	10/118780 (8.419e-05), 0 hom<br>1/120638 (8.289e-06), 0 hom<br>9/120642 (7.46e-05), 0 hom
CCF02784	Chr. 2: 179437711	326	c.73148C>T	p. S24383L	A-band	Damaging	Increased (ΔΔG = 0.64)	0	0	3/119376 (2.513e-05), 0 hom
CCF03220	Chr. 2: 179578746	92	c.26639T>G	p.F8880C	I-band	Damaging	Decreased (ΔΔG = -2.47)	0	0	0
CCF03454	Chr. 2: 179476673	268	c.50363T>C	p.I16788T	A-band	Damaging	Decreased (ΔΔG = -1.53)	0	2/5008 (0.0004)	108/117992 (0.0009153), 2 hom
CCF04477	Chr. 2: 179466465	286	c.55352G>A	p.R18451Q	A-band	Damaging	Decreased (ΔΔG = -1.47)	0	0	4/119006 (3.361e-05), 0 hom
CCF04642	Chr. 2: 179428639	326	c.82220T>C	p.I27407T	A-band	Damaging	Decreased (ΔΔG = -2.07)	G=1/A=11887 (0.000084)	1/5008 (0.0002)	4/120632 (3.316e-05), 0 hom
CCF04765	Chr. 2: 179457732	300	c.59114G>A	p.R19705H	A-band	Damaging	Decreased (ΔΔG = -0.82)	0	0	3/120538 (2.489e-05), 0 hom
CCF04880	Chr. 2: 179490087	241	c.44461A>T	p.T14821S	I-band	Damaging	Decreased (ΔΔG = -0.20)	0	0	0

Subject	Genomic position[a]	Exon	Variant	Protein alteration	Region	*In silico* predictions[b]	Protein stability[c]	NHLBI-ESP[d]	1000G[d]	ExAC[d]
CCF05254	Chr. 2: 179437076	326	c.73783G>A	p.A24595T	A-band	Damaging	Decreased (ΔΔG = -1.09)	0	0	22/120124 (0.0001831), 0 hom
CCF05417	Chr. 2: 179431234	326	c.79625G>A	p.G26542D	A-band	Damaging	Decreased (ΔΔG = -0.64)	0	0	0
CCF05501	Chr. 2: 179474599	272	c.51551T>C	p.I17184T	A-band	Damaging	Decreased (ΔΔG = -0.46)	0	0	7/120698 (5.8e-05), 0 hom
CCF06238	Chr. 2: 179633649	38	c.8914A>T	p.T2972S	I-band	Damaging	Decreased (ΔΔG = 0.03)	0	0	0
CCF06407	Chr. 2: 179501156	225	c.41298A>C	p.E13766D	I-band	Damaging	Decreased (ΔΔG = -0.15)	0	1/5008 (0.0002)	3/118050 (2.541e-05), 0 hom
CCF06705	Chr. 2: 179429212	326	c.81647G>A	p.R27216H	A-band	Damaging	Decreased (ΔΔG = -1.57)	T=1/C=11937 (0.000084)	0	5/111944 (4.467e-05), 0 hom
CCF06822	Chr. 2: 179395221	358	c.106121T>A	p.F35374Y	M-band	Damaging	Decreased (ΔΔG = -0.57)	0	0	19/120742 (0.0001574), 0 hom
CCF06990	Chr. 2: 179593302	66	c.19351G>A	p.D6451N	I-band	Damaging	Decreased (ΔΔG = -0.36)	0	0	1/118128 (8.465e-06), 0 hom
CCF07007	Chr. 2: 179600517	50	c.14656G>T	p.D4886Y	I-band	Damaging	Increased (ΔΔG = 0.39)	0	0	0
CCF07321	Chr. 2: 179403903	353	c.98759G>A	p.R32920Q	A-band	Damaging	Decreased (ΔΔG = -2.14)	0	0	6/120614 (4.975e-05), 0 hom
CCF07532	Chr. 2: 179454006<br>Chr. 2: 179434925	304<br>326	c.62446C>T<br>c.75934G>A	p.P20816S<br>p.E25312K	A-band<br>A-band	Damaging	Decreased (ΔΔG = -0.59)<br>Decreased (ΔΔG = -0.44)	0<br>0	0<br>0	0<br>3/120332 (2.493e-05), 0 hom
CCF08384	Chr. 2: 179467114	283	c.55015C>A	p.L18339M	A-band	Damaging	Decreased (ΔΔG = -0.87)	0	0	0
CCF02958	Chr. 2: 179478814	262	c.49310T>A	p.V16437D	A-band	Damaging	Decreased (ΔΔG = -1.60)	0	0	3/119324 (2.514e-05), 0 hom
CCF04542	Chr. 2: 179454746<br>Chr. 2: 179498006	304<br>233	c.61706G>A<br>c.42994G>A	p.R20569K<br>p.V14332I	A-band<br>I-band	Damaging	Decreased (ΔΔG = -1.01)<br>Decreased (ΔΔG = -0.14)	T=1/C=12045 (0.000083)<br>0	0<br>0	1/120078 (8.328e-06), 0 hom<br>0
CCF07330	Chr. 2: 179457506<br>Chr. 2: 179494976	300<br>239	c.59340G>T<br>c.44273G>A	p.R19780S<br>p.R14758Q	A-band<br>I-band	Damaging	Decreased (ΔΔG = -2.36)<br>Decreased (ΔΔG = -0.88)	0<br>0	0<br>0	0<br>3/120386 (2.492e-05), 0 hom

Subject	Genomic position[a]	Exon	Variant	Protein alteration	Region	*In silico* predictions[b]	Protein stability[c]	NHLBI-ESP[d]	1000G[d]	ExAC[d]
CCF06063	Chr. 2: 179584437	82	c.23782G>C	p.E7928Q	I-band	Damaging	Decreased (ΔΔG = -0.58)	0	0	2/120682 (1.657e-05), 0 hom
CCF04885	Chr. 2: 179571661	101	c.29062G>C	p.A9688P	I-band	Damaging	Decreased (ΔΔG = -2.24)	0	0	3/91796 (3.268e-05), 0 hom
CCF05099	Chr. 2: 179566951<br>Chr. 2: 179459286	108<br>296	c.30455G>A<br>c.57935G>A	p.R10152Q<br>p.G19312D	I-band<br>A-band	Damaging	Decreased (ΔΔG = -1.49)<br>Decreased (ΔΔG = -1.84)	T=1/C=12231 (0.000082)<br>0	0<br>0	4/120716 (3.314e-05), 2 hom<br>0
CCF05436	Chr. 2: 179408134	347	c.96566A>T	p.E32189V	A-band	Damaging	Increased (ΔΔG = -0.14)	0	0	0
CCF00102	Chr. 2: 179577926	93	c.26935A>C	p.N8979H	I-band	Damaging	Increased (ΔΔG = 0.24)	G=1/T=12263 (0.000082)	0	18/117982 (0.0001526), 0 hom
CCF06614	Chr. 2: 179473603	274	c.52135G>C	p.E17379Q	A-band	Damaging	Decreased (ΔΔG = -1.67)	0	0	0
CCF06604	Chr. 2: 179432900	326	c.77959C>G	p.P25987A	A-band	Damaging	Decreased (ΔΔG = -1.36)	0	0	1/120674 (8.287e-06), 0 hom
CCF01767	Chr. 2: 179397940	358	c.103402G>A	p.E34468K	M-band	Damaging	Decreased (ΔΔG = -0.86)	0	0	1/120682 (8.286e-06), 0 hom
CCF07575	Chr. 2: 179414752	337	c.91813T>A	p. S30605T	A-band	Damaging	Increased (ΔΔG = 0.60)	0	0	0

[a]Genomic positions are according to the human reference haploid genome sequence, hg19 and variants reported corresponding to *TTN* transcript NM_001267550.
[b]Predicted through a combination of SIFT, MutationTaster, and PolyPhen-2 (see Materials and Methods).
[c]Predicted through I-Mutant 2.0 program using the difference in the Gibbs free energy values, ΔΔG = ΔG (mutant protein) - ΔG (Wildtype protein) in Kcal/mole. The sign of ΔΔG predicts protein stability.
[d]Allele frequency data was extracted from the National Heart, Lung, and Blood Institute (NHLBI) Exome Sequencing Project (ESP) Exome Variant Server (http://evs.gs.washington.edu/EVS/) v.0.0.28, 1000 Genomes Project (http://www.internationalgenome.org) , and Exome Aggregation Consortium (ExAC), Cambridge, MA (http://exac.broadinstitute.org) all last accessed August 19, 2017.

Supplementary Table 6. *TTN* variants identified in an independent series of non-BRRS patients (n=45)

Subject	Variant[a]	Region	*In silico* predictions[b]	NHLBI-ESP[c]	1000G[c]	ExAC[c]
2283	c.25126C>T, p.P8376S	I-band	Damaging	A=1/G=11905 (0.000084)	0	13/118104 (0.0001101), 0 hom
3083	c.98867T>C, p.M32956T	A-band	Damaging	0	0	0
4367	c.94524T>G, p.D31508E	A-band	Damaging	0	0	1/112068 (8.923e-06), 0 hom
4506	c.99966G>T, p.W33322C<br>c.103471G>A, p.E34491K	A-band<br>M-band	Damaging	0<br>0	0<br>1/5008 (0.0002)	5/118208 (4.23e-05), 0 hom<br>1/120726 (8.283e-06), 0 hom
8413	c.23734G>A, p.V7912M	I-band	Damaging	0	0	0
9691	c.5132C>T, p.S1711F<br>c.43019T>C, p.I14340T	I-band<br>I-band	Damaging	0<br>0	0<br>0	2/121144 (1.651e-05), 0 hom<br>1/120294 (8.313e-06), 0 hom

[a]Genomic positions are according to the human reference haploid genome sequence, hg19 and variants reported corresponding to *TTN* transcript NM_001267550.
[b]Predicted through a combination of SIFT, MutationTaster, and PolyPhen-2 (see Materials and Methods).
[c]Allele frequency data was extracted from the National Heart, Lung, and Blood Institute (NHLBI) Exome Sequencing Project (ESP) Exome Variant Server (http://evs.gs.washington.edu/EVS/) v.0.0.28, 1000 Genomes Project (http://www.internationalgenome.org) , and Exome Aggregation Consortium (ExAC), Cambridge, MA (http://exac.broadinstitute.org) all last accessed September 3, 2017.

Supplementary Table 7. International Cowden Consortium (ICC) operational diagnostic criteria

<u>Pathognomonic</u><br>Adult Lhermitte-Duclos disease<br>Mucocutaneous lesions<br>Trichilemmomas, facial<br>Acral keratoses<br>Papillomatous papules<br>Mucosal lesions	<u>Major</u><br>Breast cancer<br>Thyroid cancer (nonmedullary)<br>Macrocephaly (i.e., ≥ 97th percentile)<br>Endometrial cancer	<u>Minor</u><br>Other thyroid lesions (eg, adenoma, multinodular goiter)<br>Mental retardation (i.e., IQ ≤ 75)<br>GI hamartomas<br>Fibrocystic breast disease<br>Lipomas<br>Fibromas<br>Genitourinary tumors (especially renal cell carcinoma)<br>Genitourinary malformations<br>Uterine fibroids
<u>Operational diagnosis in an individual</u><br>Any of following:<br>Mucocutaneous lesions alone, if ≥ six facial papules (three of which must be trichilemmomas)<br>Cutaneous facial papules and oral mucosal papillomatosis<br>Oral mucosal papillomatosis and acral keratoses<br>≥ Six palmoplantar keratoses<br>≥ Two major criteria (one of which must be macrocephaly or LDD)<br>One major and ≥ three minor criteria<br>≥ Four minor criteria		
<u>Operational diagnosis in a family where one individual is diagnostic for CS</u><br>Any one pathognomonic criterion<br>Any one major criteria ± minor criteria<br>Two minor criteria<br>History of Bannayan-Riley-Ruvalcaba syndrome		

Abbreviations: LDD, Lhermitte-Duclos Disease; CS, Cowden syndrome

Supplementary Table 8. Primer sequences and PCR cycling conditions of *TTN* I-band exons

Amplicon	Exon	Forward Primer	Reverse Primer	Annealing Temperature (°C)	Amplicon Size (bp)
1	147	CATGTGTGTTCTTATTGTGTATCTGC	GAACATCGTTAGAAGTAAATATGTAACTG	63.5	417
2	149	ACTTATATTACTGCTGTGTCTACTTG	AGTCAGAAATGACGAATGTGAAT	56.5	286
3	158-159	CGTTCATCTTGTTAGATGCCC	TGGCATGTTAGGCTTTTATAAGAG	56.5	515
4	160-161	ATTCTAGCCACTAATTTGCCTC	CCTCTGGTTGTATCAGGTTC	55.5	559
5	161-162	ATATAACACCAATGATCCACTCTGAA	AACATTAGAGGTTGTGAGAATGTATATTA	56.5	500
6	163	AAGTCCCACACATATAATATACAAAGATAA	ACTGACTCAACTATCTTTCATGTGTATAA	56.5	473
7	164-165	CCAAGAAAACAGCCGCTTA	GAAACTTCCTGTGGAACCTCA	56.5	731
8	166-167	TGGTTATATTTCCTAACTTATTGTGGTTAC	ATTTAGAAGTTACAAGAATCAACACAATC	56.5	449
9	167-168	ACCATGCTCTCAGGGTC	GCAAGAGTCAACACAGACA	62	504
10	169-171	CTGAAACACCACCTATTATGGGAA	AGGCATTATGAAGACCACTAGAA	63.2	698
11	212-213	GTATGTATATCTTACATCTCACAACTTTG	TATTTGAAGACAGAATCAGGCCTTA	62	781
12	214-215	CCCATGAAATCTAAGATGAAGCTC	AGGAATATTGTCCTGTAAATGCT	62.5	597
13	216	TGAATAATCTAATTAATAGGCAGTTCAAGT	CATACAAATGGGAACAGACATACTAC	62	374

PCR cycling conditions:

	95°C-10 minutes
37 cycles	95°C-30s<br>annealing temp°C-40s<br>72°C-1 min 20s
	72°C-10 min<br>4°C-∞

Supplementary Figure 1. Pedigree and clinical phenotypes of Family CCF01021 with an identified *TTN* variant

A

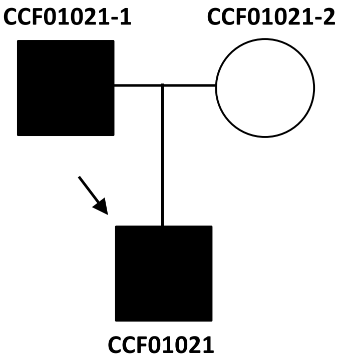

B

Genomic position	Exon	Variant	Protein alteration	Region	*In silico*[a] predictions	NHLBI-ESP	1000G	ExAC
Chr. 2: 179599265	52	c.15286T>C	p.C5096R	I-band	Damaging	0	0	0

C

Individual ID	Demographics and clinical presentation	Affected	*TTN* p.C5096R
CCF01021	31 years old, Male<br>Macrocephaly (63.5 cm), penile freckling, mental retardation, skin hemangioma, papillomatous papules, hypotonia, hydrocephalus	Yes	+
CCF01021-1	64 years old, Male<br>Macrocephaly (59.5 cm), skin hemangioma, papillomatous papules, GI polyps	Yes	+
CCF01021-2	64 years old, Female<br>No phenotype	No	-

(**A**) Pedigree of trio from Family CCF01021. Black color indicates affected status. (**B**) Characteristics of the *TTN* p.C5096R variant identified in the proband (CCF01021). (**C**) Demographic and clinical characteristics of the trio from Family CCF01021. The proband shares the same variant allele from the affected father (CCF01021-1).

[a]Predicted through a combination of SIFT, MutationTaster, and PolyPhen-2 (see Materials and Methods).

Supplementary Figure 2. Sanger sequencing for genotyping CRISPR/Cas9 genome-edited HEK293T cells for the *TTN* c.15286T>C, p.C5096R variant

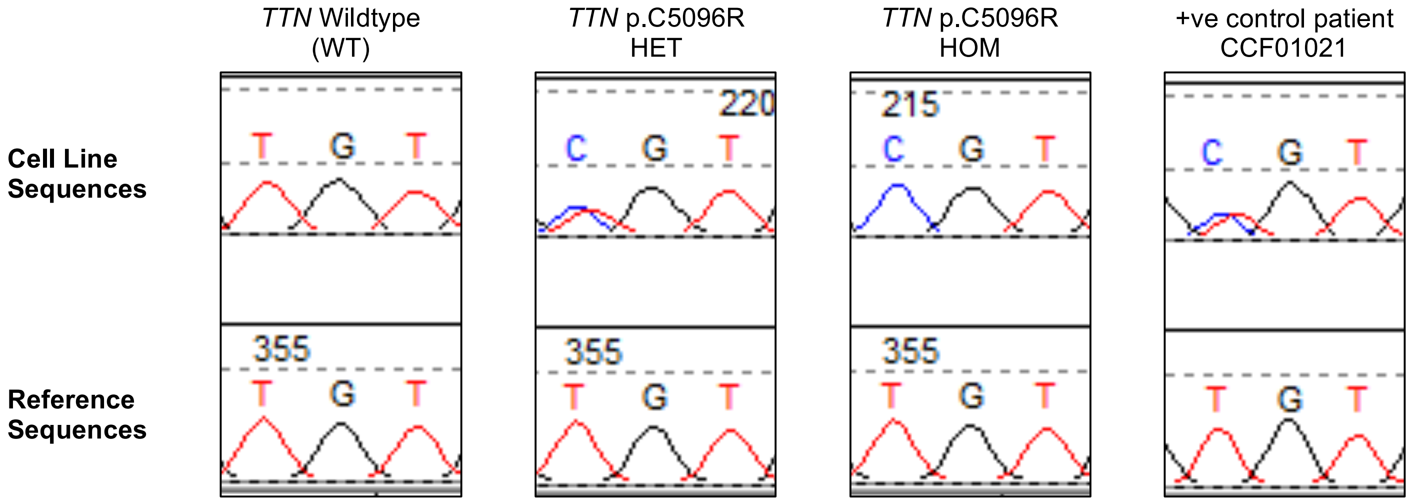

The *TTN* variant NM_001267550:c.15286T>C, p.C5096R was introduced in HEK293T cells via CRISPR/Cas9 genome editing (see Materials and Methods). Genotyping was done using PCR-based region-specific Sanger sequencing. Blood DNA from patient CCF01021 harboring the same variant was used as a positive control.

Supplementary Figure 3. Lack of contact inhibition cellular phenotype is apparent in HEK293T cells harboring the *TTN* p.C5096R variant

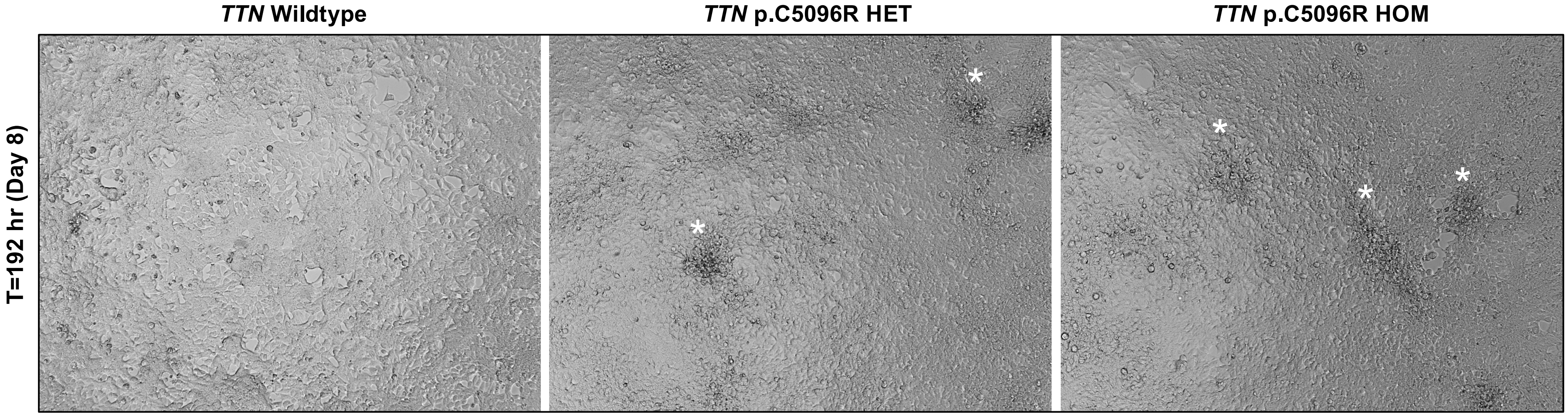

Differences in growth pattern become apparent after cells reach confluence, with mutant *TTN* p.C5096R HET and *TTN* p.C5096R HOM cells showing increased growth compared to wildtype. Foci of cells growing on top of each other are notably apparent in *TTN*-mutant cells (*). Images were taken using a Leica DMI3000B manual inverted microscope (Leica, Buffalo Grove, IL).

Supplementary Figure 4. Mutant *TTN* p.C5096R cells do not show differences in cell viability or migration

A

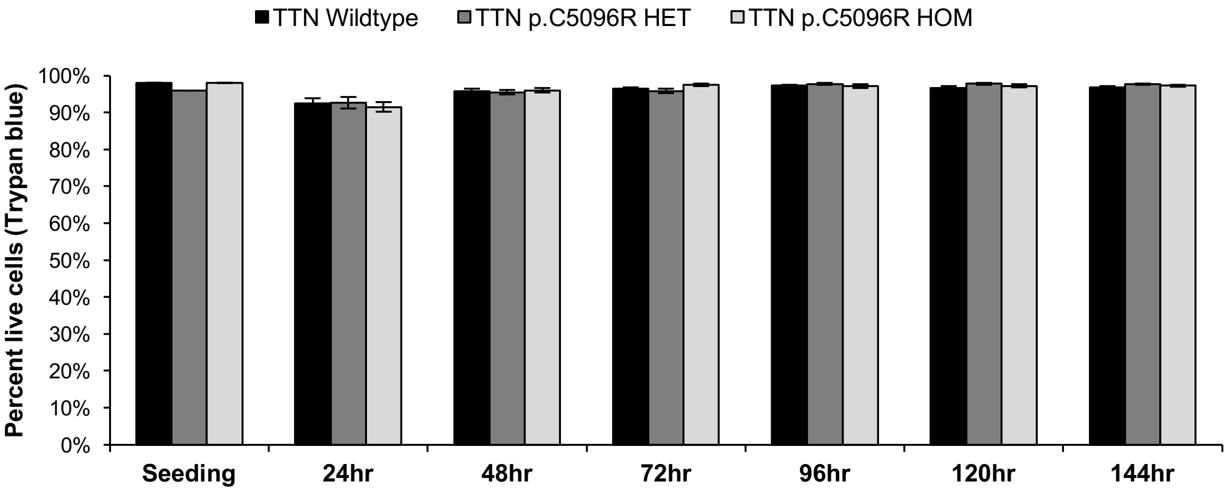

B

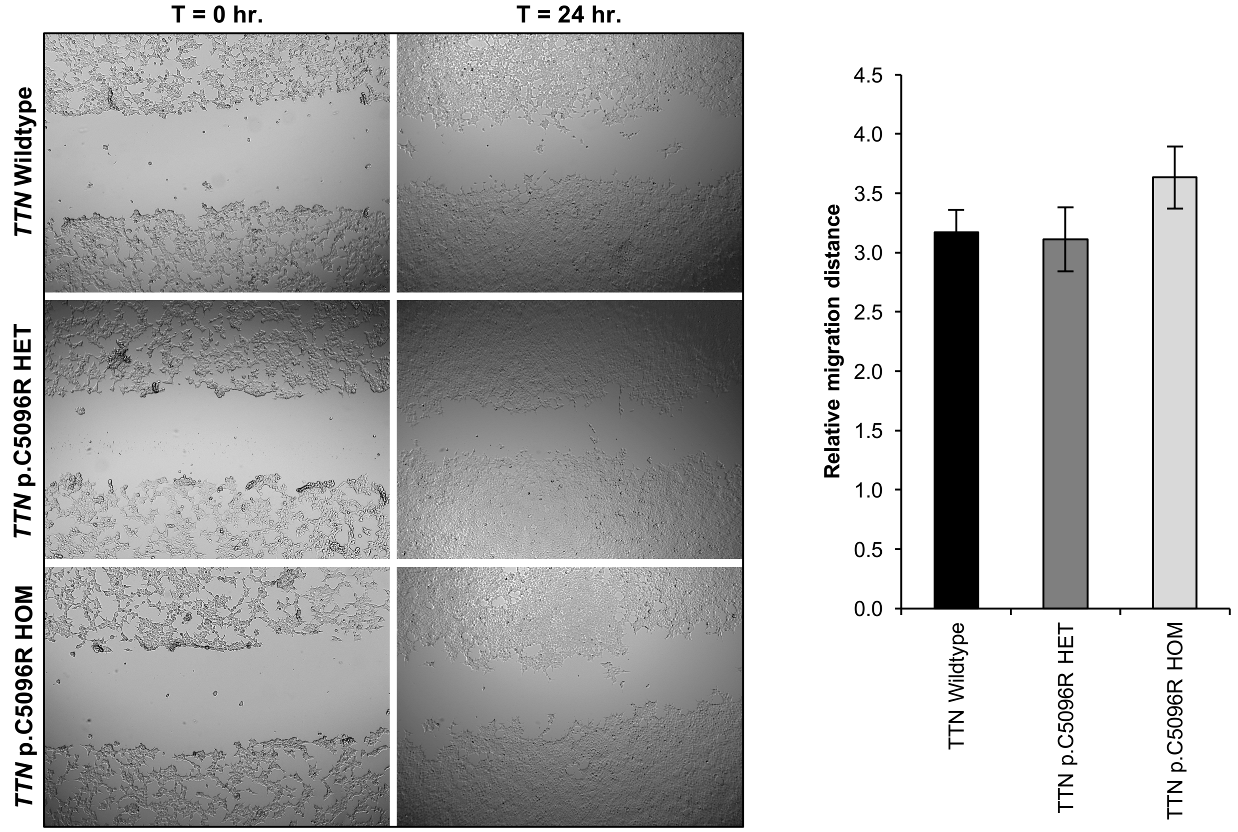

(**A**) Wildtype and mutant cells were counted for up to 144 hours post seeding. Trypan blue stain was used to count dead cells and assess viability. Experiment was done in 4 biological replicates, counted at least in triplicates at each time point. Data in figure is representative of pooled replicates; data presented as means ± SEM. (**B**) Scratches were done 24 hours after cell seeding and migration distance measured after 24 hours. Representative of 3 technical replicates; data presented as means ± SEM.

Supplementary Figure 5: Spectrum of *TTN* mutations in skeletal muscle titinopathies

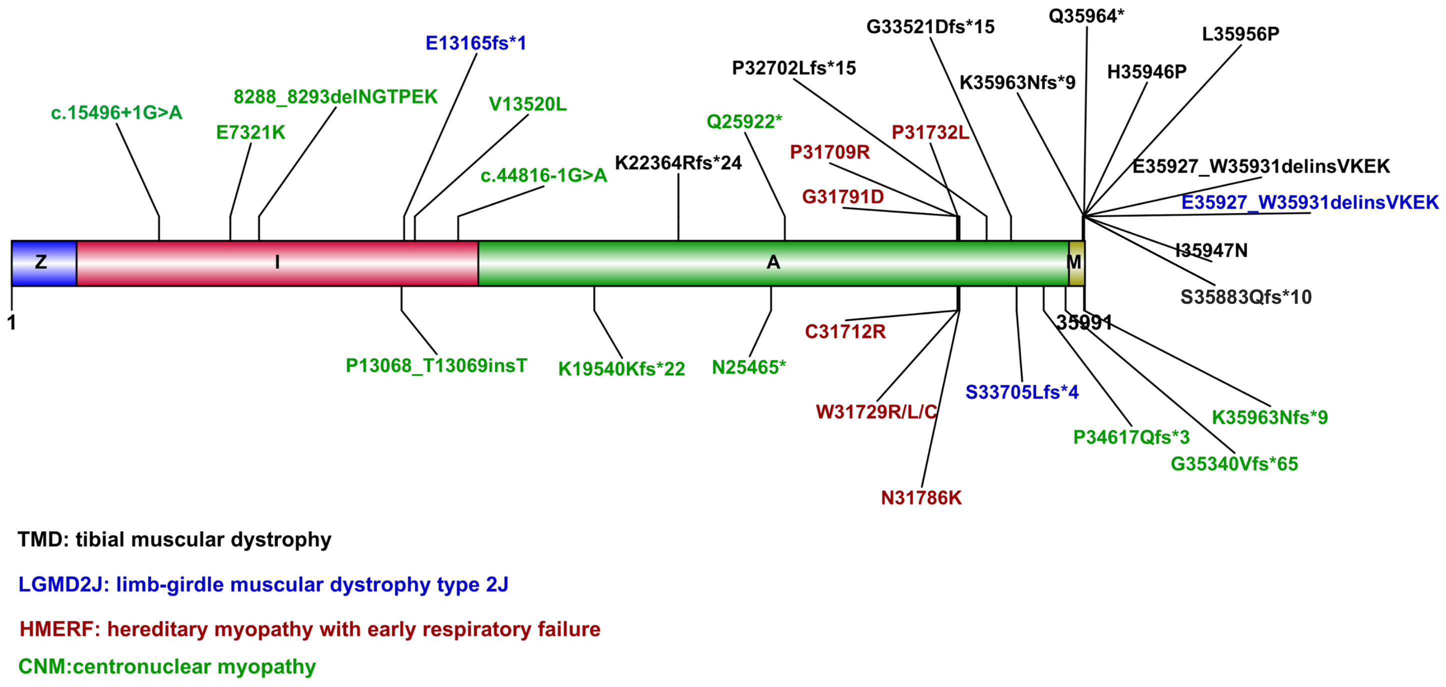

Representative of *TTN* skeletal muscle disease-causing (pathogenic or likely pathogenic) mutations extracted from Chauveau et al. *Human Mutation.* 2014;35(9):1046-1059. Mutations are depicted according to the canonical full-length IC *TTN* transcript NM_001267550. TMD is an autosomal dominant disease presenting with heterozygous *TTN* mutations. Severe cases present with homozygous or compound heterozygous mutations. All depicted *TTN* mutations in LGMD2J are homozygous or compound heterozygous. HMERF *TTN* mutations are all heterozygous except for Pro31732Leu, which was homozygous. All depicted *TTN* mutations in CNM are compound heterozygous.

Supplementary Figure 6: Spectrum of *TTN* mutations in cardiac muscle titinopathies

A. Dilated Cardiomyopathy (DCM)

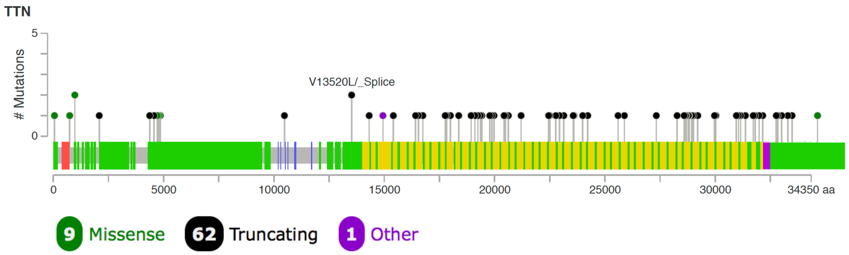

B. Arrhythmogenic Right Ventricular Cardiomyopathy (ARVC)

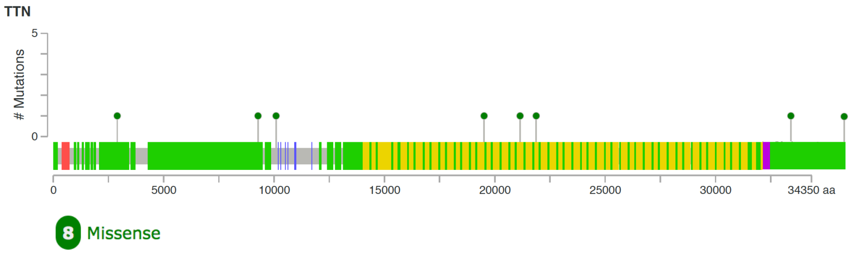

C. Hypertrophic Cardiomyopathy (HCM)

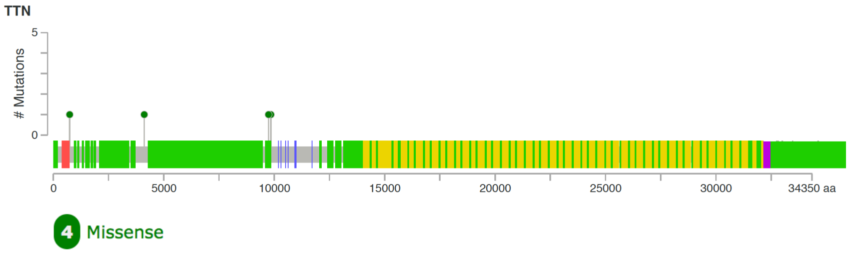

D. Restrictive Cardiomyopathy (RCM)

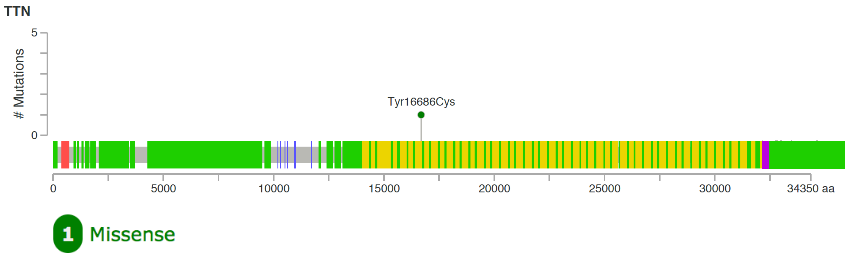

Representative of *TTN* cardiac muscle disease-causing (pathogenic or likely pathogenic) mutations extracted from Chauveau et al. *Human Mutation.* 2014;35(9):1046-1059. Mutations are depicted according to the canonical full-length IC *TTN* transcript NM_001267550. The legend term "other" in DCM (A) refers to a p.Pro14940_Thr19283dup mutation (duplication). DCM: homozygous, heterozygous, or compound heterozygous; ARVC: heterozygous or compound heterozygous; HCM: heterozygous; RCM: heterozygous. Lollipop plots were constructed using cBioPortal MutationMapper v1.0.1 (http://www.cbioportal.org/mutation_mapper.jsp).

Supplementary Figure 7: Spectrum of *TTN* mutations in combined skeletal/cardiac muscle titinopathies

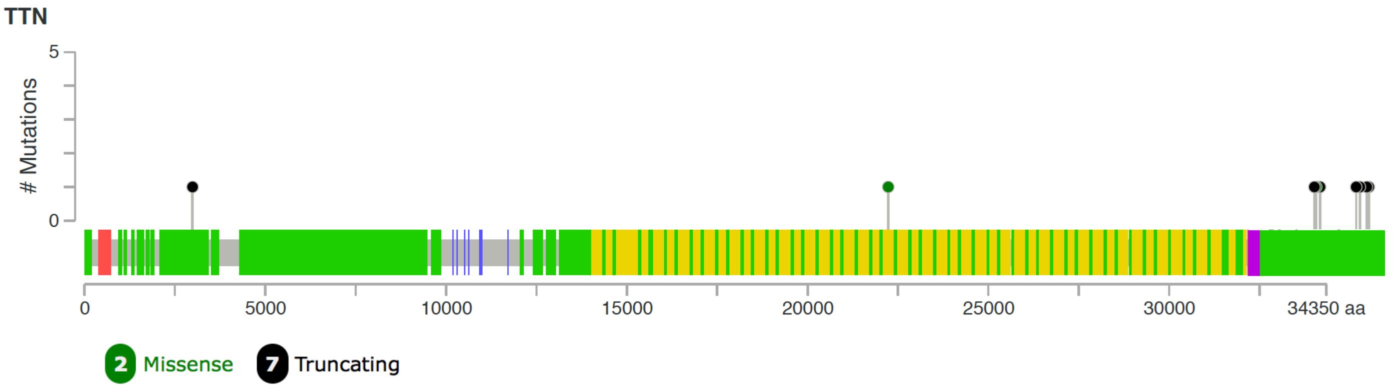

Representative of *TTN* combined skeletal/cardiac muscle disease-causing (pathogenic or likely pathogenic) mutations extracted from Chauveau et al. *Human Mutation.* 2014;35(9):1046-1059. Mutations identified in multi-minicore disease with heart disease (MmD-HD) are depicted according to the canonical full-length IC *TTN* transcript NM_001267550. All mutations are homozygous or compound heterozygous. Lollipop plots were constructed using cBioPortal MutationMapper v1.0.1 (http://www.cbioportal.org/mutation_mapper.jsp).

Supplementary Figure 8. TITIN-Interactome from BioGRID

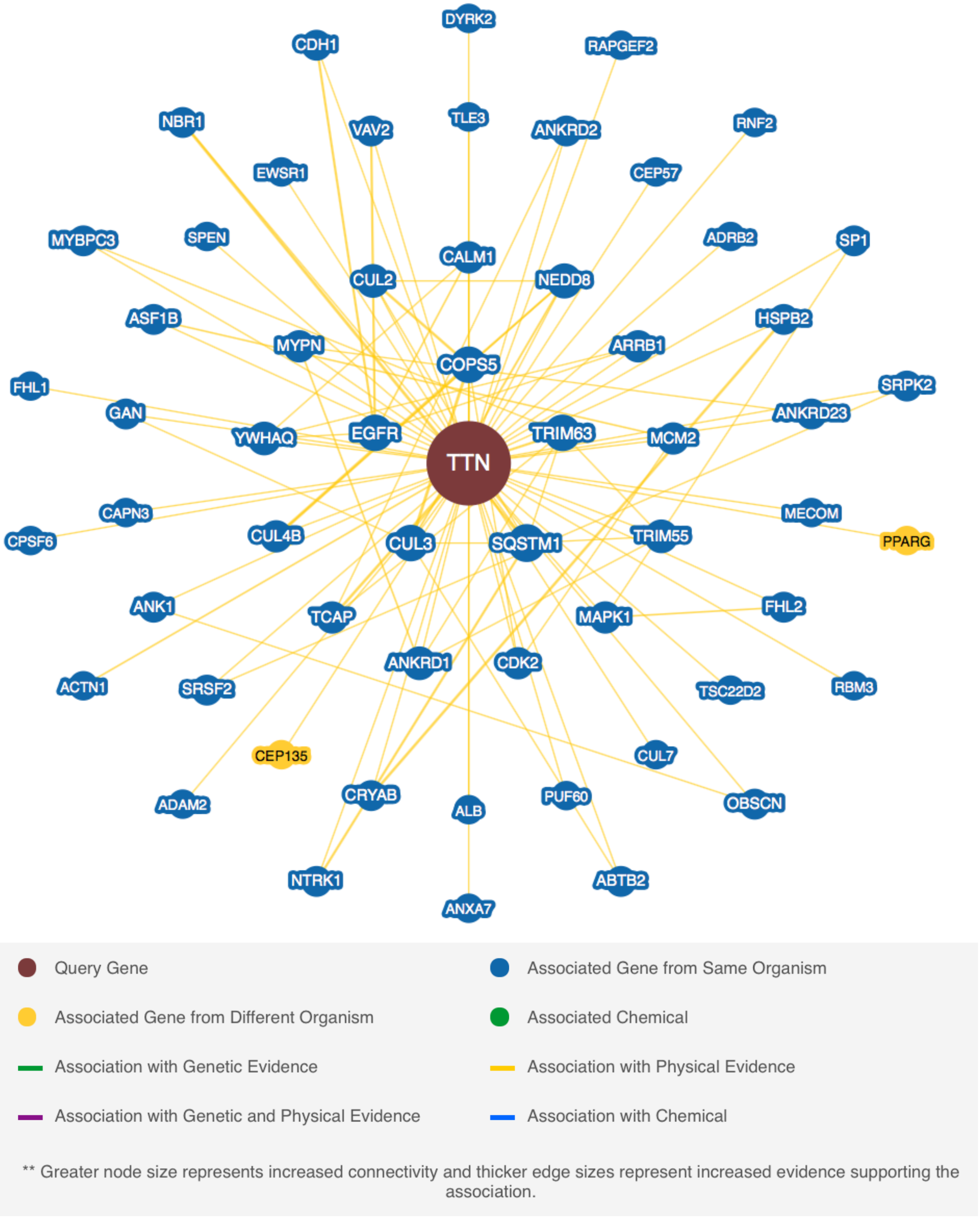

Data was extracted from the BioGRID database (https://thebiogrid.org/113124/summary/homo-sapiens/ttn.html), last accessed on November 5, 2017.

Supplementary Figure 9. Full scans of Western blots presented in the main figures

Fig. 2B

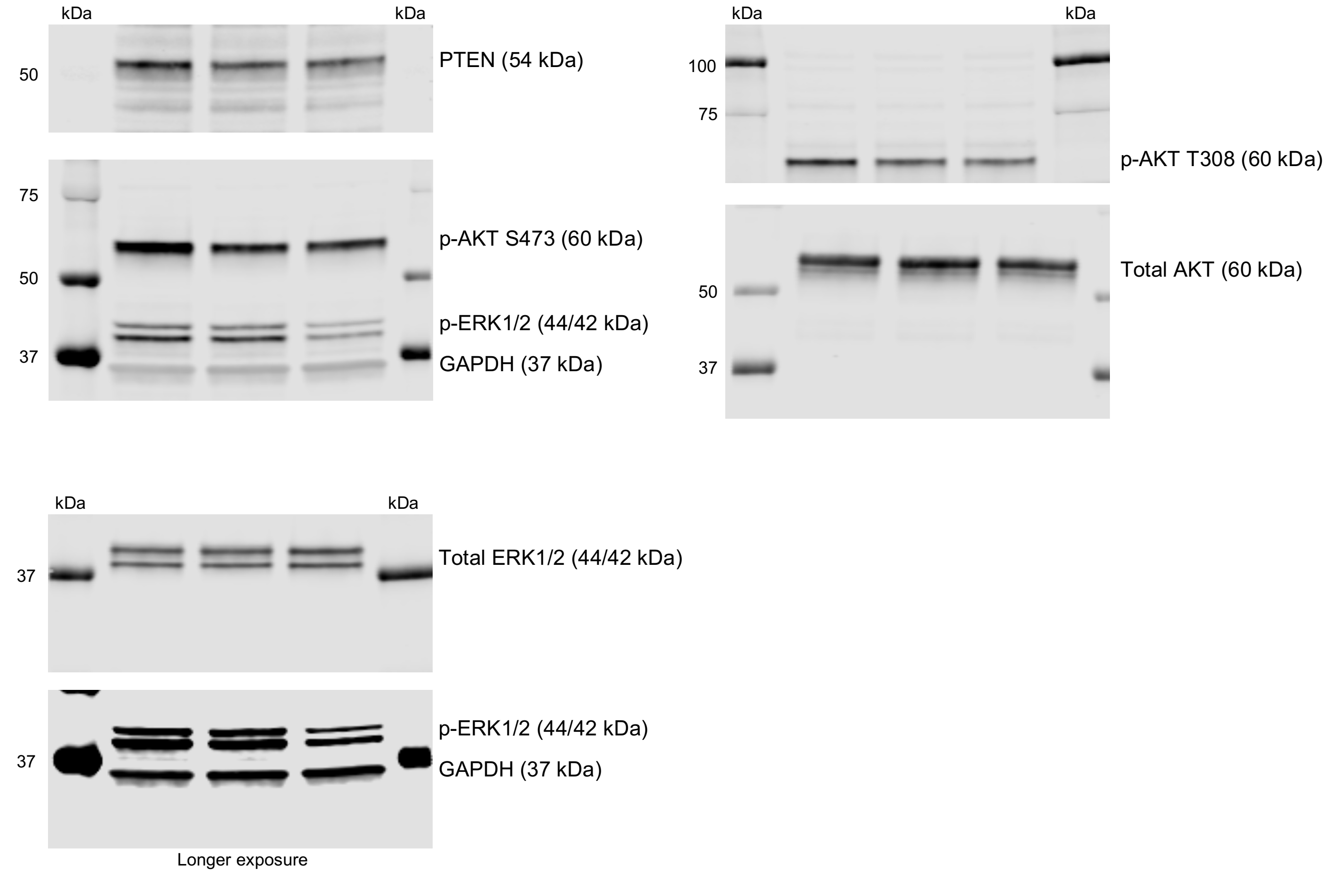

Fig. 2C

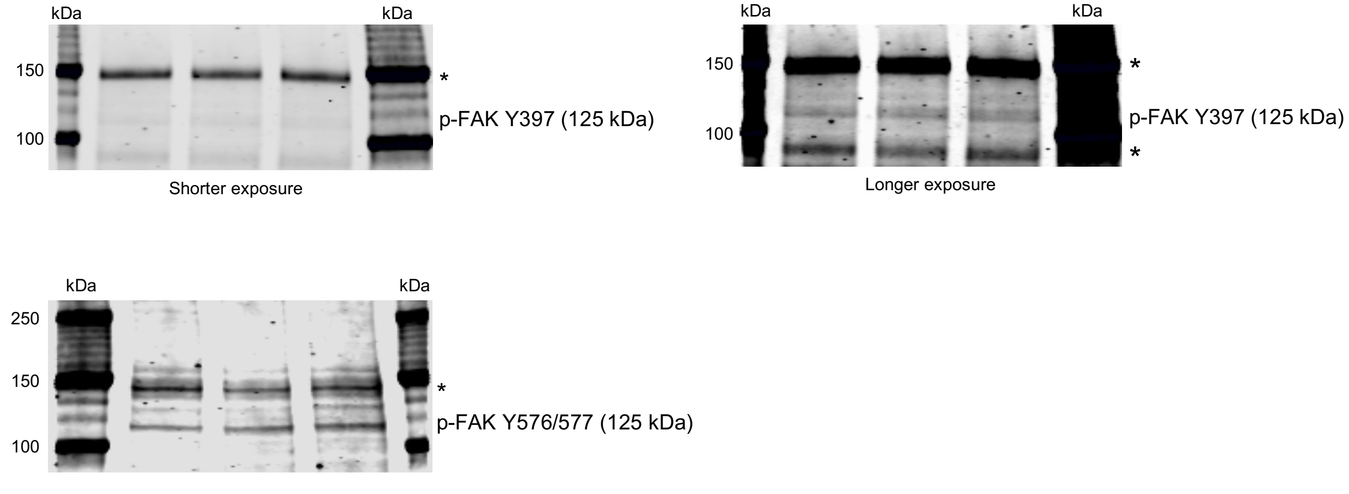

Supplementary Figure 9. Full scans of Western blots presented in the main figures (continued)

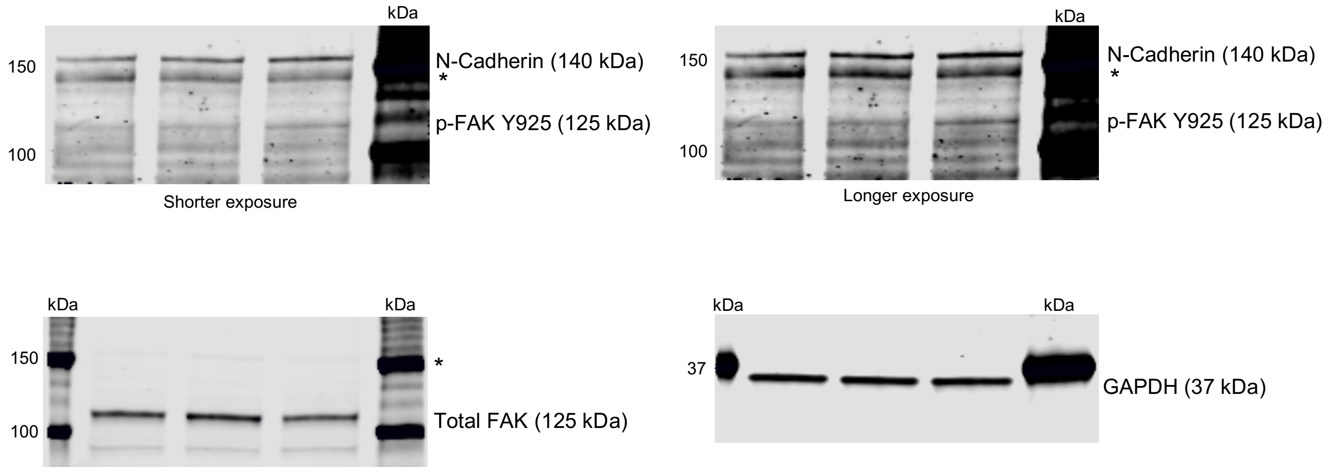

Three lanes denote protein lysates from HEK293T cells that are *TTN* wildtype, heterozygous *TTN* p.C5096R HET, and homozygous *TTN* p.C5096R HOM, respectively. Standard protein ladders (Precision Plus Protein™ Kaleidoscope™ Prestained Protein Standards BioRad #1610375) were used to denote size in kilo Daltons (kDa). Blots were scanned digitally using the Odyssey Infrared Imaging System (Li-Cor Biosciences).

For each experiment, all genotypes were run in parallel on the same gel. Antibodies include: anti-PTEN (Cascade Bioscience clone 6H2.1) at 1:2000, anti-phospho-AKT S473 (Cell Signaling #4060) at 1:1000, anti-phospho-AKT T308 (Cell Signaling #2965) at 1:1000, anti-total AKT (Cell Signaling #9272) at 1:1000, anti-phospho-ERK1/2 (Cell Signaling #9101) at 1:1000, anti-ERK1/2 (Cell Signaling #9102) at 1:1000, anti-phospho-FAK Y397 (Cell Signaling #8556) at 1:1000, anti-phospho-FAK Y576/577 (Cell Signaling #3281) at 1:1000, anti-phospho-FAK Y925 (Cell Signaling #3284) at 1:1000, anti-FAK (Cell Signaling #13009) at 1:1000, and anti-GAPDH (Cell Signaling #2118) at 1:40,000 dilutions.

Expected molecular weight in kDa of each protein is indicated in parentheses. N-Cadherin was probed for in an exploratory analysis of other relevant proteins beyond the scope of this manuscript. FAK and phospho-FAK (p-FAK) antibodies result in a non-specific band at ~140 kDa (indicated as *).
